# Assessment of Microbial Diversity in Biofilms Recovered from Endotracheal Tubes Using Culture Dependent and Independent Approaches

**DOI:** 10.1371/journal.pone.0038401

**Published:** 2012-06-05

**Authors:** Ilse Vandecandelaere, Nele Matthijs, Filip Van Nieuwerburgh, Dieter Deforce, Peter Vosters, Liesbet De Bus, Hans J. Nelis, Pieter Depuydt, Tom Coenye

**Affiliations:** 1 Laboratory of Pharmaceutical Microbiology, Ghent University, Ghent, Belgium; 2 Laboratory of Pharmaceutical Biotechnology, Ghent University, Ghent, Belgium; 3 Department of Intensive Care, Ghent University Hospital, Ghent, Belgium; Baylor College of Medicine, United States of America

## Abstract

Ventilator-associated pneumonia (VAP) is a common nosocomial infection in mechanically ventilated patients. Biofilm formation is one of the mechanisms through which the endotracheal tube (ET) facilitates bacterial contamination of the lower airways. In the present study, we analyzed the composition of the ET biofilm flora by means of culture dependent and culture independent (16 S rRNA gene clone libraries and pyrosequencing) approaches. Overall, the microbial diversity was high and members of different phylogenetic lineages were detected (Actinobacteria, beta-Proteobacteria, *Candida* spp., Clostridia, epsilon-Proteobacteria, Firmicutes, Fusobacteria and gamma-Proteobacteria). Culture dependent analysis, based on the use of selective growth media and conventional microbiological tests, resulted in the identification of typical aerobic nosocomial pathogens which are known to play a role in the development of VAP, e.g. *Staphylococcus aureus* and *Pseudomonas aeruginosa*. Other opportunistic pathogens were also identified, including *Staphylococcus epidermidis* and *Kocuria varians*. In general, there was little correlation between the results obtained by sequencing 16 S rRNA gene clone libraries and by cultivation. Pyrosequencing of PCR amplified 16 S rRNA genes of four selected samples resulted in the identification of a much wider variety of bacteria. The results from the pyrosequencing analysis suggest that these four samples were dominated by members of the normal oral flora such as *Prevotella* spp., *Peptostreptococcus* spp. and lactic acid bacteria. A combination of methods is recommended to obtain a complete picture of the microbial diversity of the ET biofilm.

## Introduction

Nosocomial pneumonia or health-care associated pneumonia is one of the most common life-threatening infections in hospitals and long-term care facilities, with an incidence ranging from 4 to 50 cases per 1000 admissions [1]. Critically ill patients requiring mechanical ventilation in the intensive care unit (ICU) constitute the group at the highest risk. Endotracheal intubation often leads to an increased occurrence of pneumonia, the so-called ventilator-associated pneumonia (VAP) [2], [3]. The prevalence of VAP among ventilated patients is 10–65%, with a mortality rate of 27–76% [2], [3]. VAP extends the ICU stay by 5–7 days and increases the length of hospital stay 2 to 3 fold, resulting in higher medical costs [2], [3]. The endotracheal tube (ET) itself is a pivotal element in the pathogenesis of VAP as it allows the direct entry of (oropharyngeal) microorganisms in the lower airways [1]–[4]. This ET is rapidly (within hours after its insertion) colonized by microorganisms that form a biofilm on its surface [2], [5], [6], [7]. This biofilm represents a significant and persistent source of pathogenic bacteria which can infect the lungs [8]. Adair *et al.* [4] reported that, in 70% ( = 14/20) of VAP patients, an identical bacterial population was found in the infected lungs and in the ET biofilms. Several mechanisms by which ET biofilm bacteria can infect the lungs were already suggested: biofilm pieces might be dispersed and passively moved towards the lungs [9], biofilms cells can be aerosolized and aspirated into the lungs and individual cells in contact with liquids can be transferred deeply into the lungs [10].

The bacterial etiology of VAP is highly diverse and distinct patterns have been identified according to the duration of intubation [11]. VAP developing within the first 2 to 5 days after intubation (early-onset VAP) is more likely caused by antibiotic-sensitive bacteria such as methicillin-sensitive *Staphylococcus aureus* and has a better prognosis. On the other hand, later occurring VAP (5 or more days after the start of mechanical ventilation) frequently involves multidrug resistant pathogens like methicillin-resistant *S. aureus* (MRSA), *Pseudomonas aeruginosa* and extended-spectrum β-lactamase (ESBL) producing Enterobacteriaceae. Late-onset VAP has been associated with a higher morbidity and mortality [4], [11]–[14].

Aerobic bacterial cultures, obtained from respiratory secretions (e.g. sputum samples) and mucosal swabs, are the mainstay of identification of the microbial etiology of VAP and guide antimicrobial treatment. In addition, surveillance cultures, taken on a regular basis, aim to detect the presence of certain high-risk pathogens colonizing the patient [13], [15], [16]. However, these cultures do not offer a complete picture of the bacterial diversity of ET biofilms. Also, isolation and identification of bacteria present in a biofilm on an implanted medical device are not always straightforward [17]. Culture dependent methods generally fail to recover and grow all biofilms cells from implanted medical devices and often lack sensitivity for the detection of chronic biofilm infections [18].

The majority of previous studies on ET biofilms focused on the identification of bronchoalveolar lavage (BAL) cultures in order to confirm VAP as recommended by the guidelines of the American Thoracic Society [11], [19], [20]. Although BAL cultures are considered as the ‘gold standard’ for the identification of respiratory pathogens, this procedure is invasive and cannot be used in some critically ill patients [20]. In addition, these studies did not identify the pool of potential pathogens present in the ET biofilm. Two recent studies [8], [19] focused on the identification of bacteria in ET biofilms. Cairns *et al.* [8] studied the diversity of 24 ET biofilms by denaturing gradient gel electrophoresis and demonstrated that the diversity of ET biofilms was high. Perkins *et al.* [19] investigated 8 ET biofilms by constructing and sequencing 16 S rRNA gene clone libraries. They identified 70% of the ET biofilm bacteria as typical members of the oral flora. As oral bacteria tend to be (facultative) anaerobic, the identification of anaerobes is desirable. In addition, an increasing number of studies suggested a potential role of anaerobes in respiratory diseases e.g cystic fibrosis [21], [22].

In the present study, ET biofilm bacteria were identified by means of cultivation and sequencing 16 S rRNA genes using clone libraries and pyrosequencing. We have applied aerobic cultivation procedures as the major ‘traditional’ pathogens known to be involved in the onset of VAP (e.g. *S*. *aureus* and *P*. *aeruginosa*) are aerobic bacteria [23]. Aerobic culture conditions are still routinely used in clinical laboratories. In addition, no guidelines for the isolation of anaerobes from ET biofilms and tracheal aspirates exists, for instance, only aerobic cultivation procedures are recommended for the analysis of sputum samples [24].

## Materials and Methods

### Setting and Patients

This study was performed in the 54-bed medical and surgical ICU of the 1056-bed Ghent University Hospital (Ghent, Belgium). All patients were older than 16 years and were intubated for at least 24 h. This study was approved by the “Ethical Committee of Ghent University Hospital” (OG017; registration number: B6702010156). A written consent was signed either by the patient or by his/her relatives. The ET samples were analyzed within 12 h after extubation. The distal 5 cm of the ET was cut off and was transferred to 10 ml 0.9% NaCl. The biofilm was removed from the tube by repeated cycles (3 times) of vortexing and sonication. 2 ml of the resulting cell suspensions were used immediately (1 ml for cultivation and 1 ml for DNA extraction). In total, 55 ET biofilms from 51 ventilated patients were analyzed. All the ET biofilms were investigated by culture dependent techniques while a selection was examined by culture independent approaches.

### Culture Dependent Identification Techniques

Diluted cell suspensions were inoculated on different isolation media. Mueller-Hinton agar (MHA) (BD, Franklin Lakes, NJ, USA) was used as a general growth medium, while Baird-Parker agar (BPA) (Oxoid, Basingstoke, UK) and mannitol salt agar (MSA) (Oxoid) were used to isolate staphylococci. Violet red bile glucose agar (VRBGA) (BD) was used for the detection of Enterobacteriaceae, while cetrimide agar (CA) (BD) was used for the isolation of *Pseudomonas* spp. The inoculated media were incubated aerobically at 37°C for several days. Pure cultures were obtained from all isolation media and were stored at −80°C using MicroBank (ProLab, Neston, UK) vials. Conventional microbiological tests (Gram staining, microscopical analysis, oxidase and catalase tests) were performed on all isolates using standard procedures.

#### Identification of Gram-negative bacteria

Gram-negative, oxidase-negative bacilli were inoculated on Kligler’s medium (LabM, Bury, UK) in order to determine if they belong to the Enterobacteriaceae. All putative Enterobacteriaceae were further identified by API 20 E (BioMérieux, Marcy l’Etoile, France) according to the manufacturer’s instructions. Gram-negative, oxidase-positive bacilli which produce a greenish pigment on MHA plates were considered to be *Pseudomonas* spp. These isolates were inoculated on MHA and CA plates that were incubated overnight at 42°C and 37°C, respectively. The identification was confirmed by analyzing a selection of isolates by API 20 NE (BioMérieux). Gram-negative, oxidase-negative, non-Enterobacteriaceae were identified by API 20 NE (BioMérieux).

#### Identification of Gram-positive bacteria

The catalase test differentiated between members of the genera *Staphylococcus* and *Micrococcus* on the one hand and lactic acid bacteria on the other hand. Gram-positive, catalase-positive cocci were tested for the presence of coagulase (BD) and DNase (Oxoid) enzymes. In addition, *Staphylococcus* spp. and *Micrococcus* spp. were differentiated by the lysostaphin test (Sigma, Bornem, Belgium). A multiplex PCR to differentiate *S. aureus* from *Staphylococcus epidermidis* was performed as previously described [25]. The PCR products were separated by electrophoresis (1 h at 100 V) on a 1% agarose gel (Invitrogen, Merelbeke, Belgium) containing 0.01% GelRed (VWR, Leuven, Belgium). The presence of a fragment of 124 bp identified the isolate as *S. epidermidis*, while the presence of a fragment of 108 bp identified it as *S. aureus*. Micrococci and isolates that were not identified by the multiplex PCR were inoculated on a API Staph strip (BioMérieux) according to the manufacturer’s instructions. Gram-positive, catalase-negative and oxidase-negative cocci were identified by API Strep (BioMérieux). Isolates that were not identified by the methods listed above were identified by 16 S rRNA gene sequence analysis as described elsewhere [26]. Partial 16 S rRNA sequences (covering hypervariable regions V1 to V3) were obtained using conserved primers i.e. 27F (5′ AGA GTT TGA TCC TGG CTG AG 3′) and 519R (5′ GTA TTA CCG CGG CTG CTG 3′) [26]. The PCR products were separated by agarose gel electrophoresis. Thereafter, amplicons with the proper length (500 bp) were sequenced according to the manufacturer’s protocol (Applied BioSystems, Carlsbad, CA, USA) and as described earlier [26]. The amplicons were separated by capillary gel electrophoresis (Applied BioSystems) and data were analyzed using Sequence Scanner v1.0 (Applied BioSystems) and BioEdit [27]. The BLAST program (http://blast.ncbi.nlm.nih.gov/blast) was used to find the most similar sequences in public databases. As we generated partial sequences (500 bp), we used stringent identification criteria, namely only sequences with at least 99% sequence homology with type strains in public databases were included in this study.

#### Identification of yeasts

Yeast cells were identified by inoculation on the chromogenic CHROMAgar *Candida* Medium (BD) according to the manufacturer’s instructions. After 24 h incubation at 37°C, the yeasts were identified by the color of colony (*Candida albicans* produced green colonies).

### Culture Independent Identification Techniques

#### Construction of 16 S rRNA gene clone libraries

For the 20 first ETs, 16 S rRNA gene clone libraries were constructed. DNA was extracted directly from the ET biofilm as described previously [28] and 16 S rRNA genes were amplified using the same primers as in the culture dependent identification techniques (i.e. 27F and 519R) [26]. Partial PCR amplified 16 S rRNA genes (500 bp) were ligated into pGEM-T Easy Vectors (Promega, Leiden, The Netherlands) according to the manufacturer’s instructions. Clones were picked up and plasmids were extracted [29]. The inserted amplicons were amplified by PCR using a commercial forward T7 promotor primer (Promega) and a reverse SP6 promotor primer (Promega) according to the manufacturer’s instructions. The PCR amplicons were separated by gel electrophoresis (2.5% agarose, 1 h at 100V) and only fragments with the proper length (700 bp) were selected for sequencing (as described earlier) [26]. Chimeric sequences were removed using the bio-informatics toolkit (www.bioinformatics-toolkit.org). The RDP software platform (http://rdp.cme.msu.edu/) was used to define operational taxonomic units (OTUs) in each sample with a cutoff of 99% sequence similarity. Subsequently, the BLAST program was used to find to most similar sequences in public databases; only sequences with at least 99% sequence similarity to type strains in public databases were incorporated in our study.

#### Pyrosequencing of PCR amplified 16 S rRNA genes

The diversity of four samples was also investigated by Roche GS FLX second generation sequencing (Roche, Brussel, Belgium) of PCR amplified 16 S rRNA genes. Samples were selected based on the results obtained by the culture dependent analyses and we included a sample apparently dominated by Enterobacteriaceae (E1), a sample apparently dominated by *P*. *aeruginosa* (E13) and two samples with a more diverse composition (E4 and E17). We amplified the same regions (V1 to V3) of the 16 S rRNA genes as we did in the culture dependent identification methods and the construction of clone libraries [26]. Fusion primers based on 27F and 519R primer sequences (see earlier) and containing the GS FLX titanium adaptor sequences and a multiplex identifier, were designed according to the manufacturer’s instructions (IDT, Leuven, Belgium). An amplification PCR was performed on the DNA extracted directly from the samples according to the manufacturer’s instructions (Roche). The quality and quantity of the amplicons were measured by electrophoresis according to the manufacturer’s protocols (Agilent Technologies, Santa Clara, CA, USA). Thereafter, an emulsion-based clonal amplification was performed according to the Roche GS FLX titanium series emPCR and the libraries were sequenced according to the Roche GS FLX titanium sequencing method manual – version October 2009. The tags and the primers were removed from the reads and the resulting sequences were analyzed using the ribosomal database project (RDP) pyrosequencing pipeline (http://pyro.cme.msu.edu/). In addition, chimeric sequences were removed from the dataset using the bioinformatics toolkit (www.bioinformatics-toolkit.org). OTUs were defined (per sample) as sequences with at least 99% sequence similarity. One representative sequence per OTU was selected using the dereplication tool of the RDP pyrosequencing pipeline and was identified using the BLAST program. Only sequences with at least 99% sequence similarity to type strains and which appeared more than 10 times in our dataset were considered.

### Statistical Analysis

The Simpson index of diversity (1-D) [30] was calculated in order to compare the bacterial diversity of ET biofilms as revealed by culture dependent and culture independent techniques (D = ∑ (n*_i_*/N)^2^ where n*_i_* is the number of individuals belonging to species *i* in any given sample and N is the total number of individuals present in any given sample). A Simpson diversity index close to 1 means that the sample is highly diverse. In addition, Good’s coverage coefficient [31] was computed in order to determine the coverage of culture dependent and culture independent methods (C = [1– (p*_i_*/N)]×100; where p*_i_* is the number of unique phylotypes in any given sample and N is the total number of individuals in any given sample). A Good’s coverage coefficient close to 100 represents a completely identified sample. The Mann-Whitney U test was used to compare the indexes of the different samples. The SPSS software was used (version 17.0; SPSS Inc., Chicago, IL, USA) and the statistical significance was defined as a p value less than 0.05. In addition, collector’s curves (plotting the number of species as a function of the number of clones/pyrosequences investigated) were constructed. A clustering analysis (Unifrac) was performed on 16 S rRNA gene sequences of the clones and the isolates using the mothur software [32]. Four groups were considered: patients with VAP, patients without VAP, patients who were intubated less than 5 days and those which were intubated more than 5 days. In addition, the identification results of the culture dependent analyses of ET biofilms from patients with or without VAP were compared and statistically examined by the chi square test using the SPSS software (SPSS Inc.). The tests were two-tailed and statistical significance was set as a p value less than 0.05.

### Accession Numbers

The GenBank accession numbers of selected 16 S rRNA gene sequences from isolates are JN652258 to JN652279. The GenBank accession numbers of selected 16 S rRNA gene sequences of clones are JN652280 to JN652293. The pyrosequences were submitted to GenBank using the Sequence Read Archive tool (project submission number: SRA045823.2).

## Results

### Identification by Culture Dependent Analysis

573 isolates were picked up from the 55 ET examined; 121 from MHA, 174 from BPA, 137 from MSA, 106 from VRBGA and 35 from CA. Nineteen isolates failed to grow after the first round of subculturing. Thus, 554 pure cultures were available for further study, including 334 Gram-positive bacteria, 157 Gram-negative bacteria and 65 yeasts.

Catalase-positive, Gram-positive isolates (n = 306) were screened for the presence of coagulase and DNase activity and were further identified by multiplex PCR, API Staph and 16 S rRNA gene sequence analysis. In this way, 71 coagulase/DNase positive isolates were identified as *S. aureus* (12 ETs). 237 isolates were coagulase/DNase negative and 40 of them were identified as *Micrococcus luteus* (16 ETs) and 3 as *Kocuria varians* (3 ETs). However, most of the ‘coagulase-negative staphylococci’ (CoNS) were identified as *S. epidermidis* (n = 131). *S*. *epidermidis* was the most frequently encountered organism as it was present on 33 of the 55 ETs (Table 1). Also, *Staphylococcus saprophyticus* (n = 29; 13 ETs), *Staphylococcus haemolyticus* (n = 7; 5 ETs), *Staphylococcus hominis* (n = 5; 4 ETs) and *Staphylococcus xylosus* (n = 6; 5 ETs) were encountered (Table 1). Other staphylococci (6 species) represented only a minor fraction and were found on 1 to 3 ETs (Table 1). Fourteen isolates showed no catalase activity and were identified as *Gemella morbillorum* (n = 6; 2 ETs), *Enterococcus faecium* (n = 5; 4 ETs) and *Lactococcus lactis* (n = 3; 2 ETs). Seven Gram-positive rods were identified as *Bacillus simplex* (4 ETs), 3 as *Rhodococcus corynebacterioides* (3 ETs) and 1 as *Leifsonia aquatica*. One isolate had a mycelium-like cell morphology and was identified as *Streptomyces* spp. (Table 1).

**Table 1:** Summary of the culture dependent identification results.

	Identification	#Isolates	#ETs	%ETs
Staphylococci	*Staphylococcus epidermidis*	131	33	60%
	*Staphylococcus aureus*	71	12	22%
	*Staphylococcus saprophyticus*	29	13	24%
	*Staphylococcus haemolyticus*	7	5	9%
	*Staphylococcus hominis*	5	4	7%
	*Staphylococcus xylosus*	6	5	9%
	Other staphylococci (*Staphylococcus capitis*, *Staphylococcus cohnii*, *Staphylococcus hyicus*, *Staphylococcus lentus*, *Staphylococcus pasteurii*, *Staphylococcus warneri*)	14	1 to 3	
Lactic acid bacteria	*Enterococcus faecium*	5	4	7%
	Other lactic acid bacteria (*Gemella morbillorum*, *Lactococcus lactis*)	9	1 to 3	
Other Gram-positive	*Bacillus simplex*	7	4	7%
bacteria	*Micrococcus luteus*	40	16	29%
	Other Gram-positive bacteria (*Kocuria varians*, *Rhodococcus corynebacterioides*, *Leifsonia* *aquatica*, *Streptomyces* spp.)	8	1 to 3	
Enterobacteriaceae	*Klebsiella pneumoniae*	31	4	7%
	*Enterobacter aerogenes*	11	4	7%
	*Escherichia coli*	12	6	11%
	Other Enterobacteriaceae (*Enterobacter sakazakii*, *Raoultella ornithinolytica*, *Shigella* spp.,*Klebsiella oxytoca*, *Hafnia alvei*)	25	1 to 3	
Other Gram-negative	*Pseudomonas aeruginosa*	52	6	11%
bacteria	Other Gram-negative bacteria (*Stenotrophomonas maltophilia*, *Sphingomonas paucimobilis*, *Acinetobacter lwoffii*, *Photobacterium damselae*, *Myroides odoratus*, *Aeromonas sobria*,*Pasteurella* spp., *Moraxella* spp.)	26	1 to 3	
Yeasts	*Candida albicans*.	60	10	18%
	*Candida* spp	5	5	9%

The number of isolates per species and the number and percentage of ETs on which they were found are also given.

Ninety-three (93/157) of the rod-shaped, Gram-negative isolates gave a negative result for the oxidase test and 79 of them fermented glucose (29/79 isolates also fermented lactose). These isolates were considered to be Enterobacteriaceae. Thirty-one isolates were identified as *Klebsiella pneumoniae* (4 ETs), 11 as *Enterobacter aerogenes* (4 ETs) and 12 as *Escherichia coli* (6 ETs) (Table 1). Twenty-five Enterobacteriaceae (5 species) represented only a minor fraction of the isolates and were found on 1 to 3 ETs (Table 1). Fourteen isolates neither used glucose nor lactose and were identified as *Stenotrophomonas maltophilia* (n = 10; 2 ETs), *Sphingomonas paucimobilis* (n = 2; 2 ETs) and *Acinetobacter lwoffii* (n = 2; 2 ETs). Fifty-two isolates were rod-shaped and oxidase-positive, and produced pigment on MHA plates. Identification results demonstrated that these isolates belong to *P. aeruginosa* (6 ETs). In contrast, 8 of the 60 oxidase-positive, rod-shaped Gram-negative isolates did not produce a greenish-brown pigment on MHA and were identified as *Photobacterium damselae* (n = 3; 3 ETs), *Myroides odoratus* (n = 3; 2 ETs), *Aeromonas sobria* (n = 1; 1 ET) and *Pasteurella* spp. (n = 1; 1 ET). Four Gram-negative bacteria had a coccoid cell morphology and were identified as *Moraxella* spp. (2 ETs) (Table 1).

Sixty-five isolates showed a yeast-like morphology and 60 of them were identified as *Candida albicans* (10 ETs); 5 isolates could only be identified to the genus level (*Candida* spp.) (5 ETs) (Table 1).

Four patients were extubated and subsequently re-intubated in the course of our study and both samples were investigated (Table 2). Some species were present on both ETs from the same patient, i.e. *S*. *epidermidis* occurred in both E25 and E32 (patient 24), *E*. *coli* in E26 and E29 (patient 25) and *C*. *albicans* in E34 and E36 (patient 31). However, overall there was little similarity between both samples from any given patient (Table 2).

**Table 2:** Identification results of the ET biofilm flora of patients from whom two tubes were investigated.

Patient	Sample	Species recovered
1	E01	*E*. *aerogenes*, *E*. *coli*, *R*. *ornithinolytica*, *S*. *lentus*, *S*. *capitis*, *C*. *albicans*
	E03	*R*. *planticola*, *S*. *epidermidis*, *S*. *xylosus*, *S*. *warneri*
24	E25	*S* . *epidermidis*, *S*. *saprophyticus*, *C*. *albicans*, *Candida* spp.
	E32	*S* . *epidermidis*, *Candida* spp.
25	E26	*E* . *coli*, *S*. *saprophyticus*, *S*. *epidermidis*
	E29	*E* . *coli*
32	E34	*C* . *albicans*, *M*. *luteus*
	E36	*C* . *albicans*, *S*. *maltophilia*

Species recovered in both samples from a given patient are underlined.

### Identification by Culture Independent Analyses

DNA was directly extracted from ET biofilms and 16 S rRNA genes were amplified. 20 samples were randomly selected for the construction of clone libraries and 4 samples were selected for pyrosequencing analysis. We identified partial 16 S rRNA gene sequences to the species level, therefore, we used a cutoff of 99% sequence similarity to type strains in public databases. Although, we are aware that these identifications are not definitive, we believe that this is the only way to make a good comparison between the different identification methods used in the present study.

973 clones were obtained and 400 clones were randomly selected for sequence analysis. 13 chimeric sequences were removed and in this way, 387 clones (40%) were identified by sequencing (average sequence length: 500 bp). Nearly one third (n = 123) of the sequenced clones belonged to the Enterobacteriaceae, including *E. aerogenes* (n = 76; 6 ETs), *Enterobacter* spp. (n = 26; 5 ETs) and *Klebsiella* spp. (n = 21; 4 ETs). Lactic acid bacteria (n = 86) constituted the second most abundant group, comprising *Gemella haemolysans* (n = 45; 3 ETs), *Lactococcus fermentum* (n = 17; 2 ETs), *Streptococcus pneumoniae* (n = 17; 3 ETs) and *E*. *faecium* (n = 7; 1 ET). Other frequently recovered sequences belonged to *P*. *aeruginosa* (n = 52; 5 ETs), *S*. *epidermidis* (n = 17; 4 ETs), *Leptotrichia* spp. (n = 12; 2 ETs) and *Bacillus cereus* (n = 10; 1 ET). Minor fractions of the clones were identified as *S*. *haemolyticus* (n = 9; 2 ETs), *M. luteus* (n = 8; 3 ETs), *Alcaligenes* spp. (n = 7; 2 ETs), *Photobacterium* spp. (n = 4; 1 ET), *S*. *aureus* (n = 3; 1 ET) and *Actinomyces odontolyticus* (n = 3; 2 ET) (Table S2).

By pyrosequencing, 139968 reads were obtained (after trimming, quality control and the removal of chimeric sequences). Sequences with at least 99% sequence similarity with known taxa and of which more than 10 copies were present, were included in our study. In this way, 106814 reads (average sequence length: 480 bp; 48455 reads from E1, 31780 from E4, 14642 from E13 and 11937 from E17) representing 4026 OTUs were obtained (Table S2).

**Figure 1:**
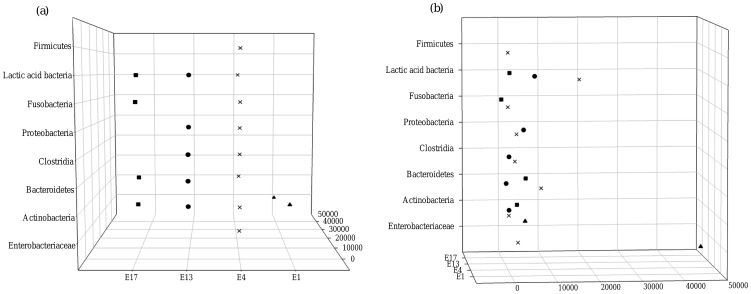
Representation of the most dominant bacterial groups identified by pyrosequencing (**a**)**.** Rotation of Figure 1a. This graph shows that most sequences appeared less than 10000 times (**b**). E1: triangles; E4: crosses; E13: circles; E17: squares.

Pyrosequencing analysis of E1 demonstrated the predominance of Enterobacteriaceae (16445 identified reads) including *E*. *aerogenes* (n = 13323) and *Serratia marcescens* (n = 2904). Remarkably, more than half of the reads from sample E1 (28707/48455) were identified as members of the Enterobacteriaceae but could not be identified to the species level. Also, *Mycoplasma salivarium* (n = 3293) was present in sample E1 (Fig. 1, Table S2). Sample E4 was more diverse as pyrosequencing showed the presence of 33 different species belonging to the Actinobacteria (2 species), Bacteroidetes (5 species), Clostridia (6 species), Enterobacteriaceae (5 species), β-Proteobacteria (1 species), Fusobacteria (2 species), γ-Proteobacteria (2 species), ε-Proteobacteria (1 species), Firmicutes (1 species) and lactic acid bacteria (8 species). Pyrosequencing also demonstrated that the diversity of sample E13 was quite high; species belonging to Actinobacteria (e.g. *Rothia mucilaginosa*: 647 reads), Bacteroidetes (e.g. *Prevotella histicola*: 396 reads), Clostridia (e.g. *Veillonella parvula*: 1106 reads), lactic acid bacteria (e.g. *Granulicatella adiacens*: 4229 reads) and γ-Proteobacteria (*P*. *aeruginosa*: 4675 reads) were identified. Finally, analysis of the pyrosquences of sample E17 revealed the presence of Actinobacteria (e.g. *Atopobium rimae*), Fusobacteria (e.g. *Fusobacterium nucleatum*), Bacteroidetes (e.g. *Prevotella oris*) and lactic acid bacteria (e.g. *Lactobacillus crispatus*) (Fig. 1, Table S2).

### Data Analysis

Simpson diversity indexes (1–D) were calculated for cultivation, clone libraries and pyrosequencing experiments. Twelve ET biofilms, studied by cultivation, had a very low bacterial diversity (1-D = 0) as only one bacterial species was identified (i.e. E21, E23, E27, E28, E29, E31, E36, E38, E39, E45, E48 and E51) (Table S1, Table S3). Other samples were more diverse e.g. E16 had a 1-D value of 0.87 (Table S1, Table S3). The number of species recovered per ET biofilm by culture dependent analysis varied from 1 to 12 (E16) and on average, 3 different species were found per ET biofilm. The majority of ET biofilms contained 1 (n = 12), 2 (n = 13) or 3 (n = 8) species; 19 ET biofilms contained 4 or more species (4, n = 6; 5, n = 5; 6 or more, n = 8). cE6 was the only clone library in which only a single species was identified (1-D = 0). Two or more species (max. 6) were identified in the other clone libraries, resulting in a 1-D index ranging between 0.12 and 0.59 (Table S3). On average, sequencing of the clone libraries revealed 3 different species per ET biofilm. The Simpson diversity indexes did not differ significantly between both methods (cultivation and sequencing of the clone libraries) (p>0.05). In contrast, the Simpson diversity indexes for the 4 samples studied by pyrosequencing ranged between 0.51 (E1) and 0.87 (E4) and were significantly higher (p<0.05) than the 1-D values of cultivation and clone libraries (Table S3). The number of species per ET biofilm detected by pyrosequencing was high: 9 species in sample E1, 33 species in sample E4, 14 species in sample E13 and 18 species in sample E17.

**Figure 2:**
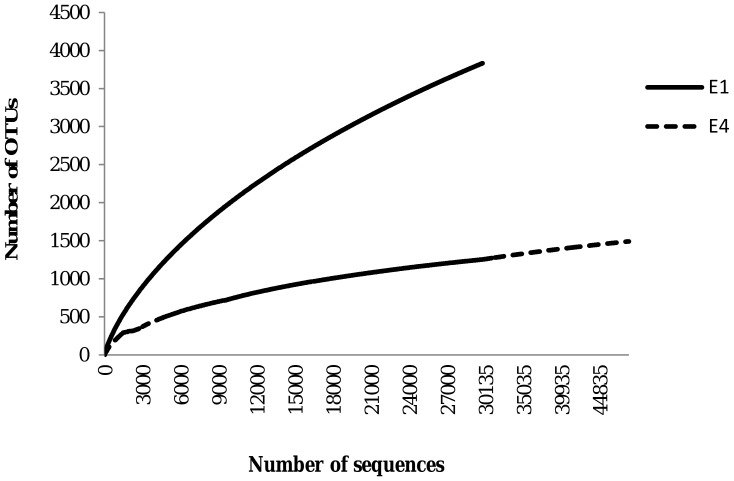
Collector’s curves of two selected pyrosequencing experiments (E1 and E4). The cut-off for OTU delineation was 99% sequence similarity. OTU: operational taxonomic unit.

Good’s coverage coefficient ranged from 0 to 96 for the culture dependent identification while the coefficient was much less variable (ranging from 90 to 98) for the clone libraries (Table S3). Plotting the number of species identified as a function of the number of clones investigated (collector’s curve), indicated that the screen was saturated and that the diversity observed is likely to be a good estimate of the real diversity (Table S4). In contrast, collector’s curves based on pyrosequencing data showed a steeper slope. However, Good’s coverage coefficient was high for the 4 samples investigated by pyrosequencing (ranging from 89 to 99), indicating that pyrosequencing reflected the bacterial diversity accurately (Fig. 2, Fig. S1, Table S3).

Clustering analysis (Unifrac) of 16 S rRNA gene sequences of clones and isolates demonstrated that there were no significant differences between the bacterial populations of patients with and without VAP (p = 0.66) and between patients which were intubated more than 5 days and those which were intubated less than 5 days (p = 0.51).

## Discussion

### Microbial Diversity of ET Biofilms as Revealed by Culture Dependent Techniques

*S*. *epidermidis* was the most frequently encountered organism and this organism is increasingly recovered as the causative agent of nosocomial infections [33], [34]. *M*. *luteus* was also found; it is considered a non-pathogenic skin bacterium, but it has caused various infections in immunocompromised patients [35], [36], [37]. *S*. *aureus* was found in 22% of ET biofilms; it is frequently involved in the development of late-onset VAP [23]. *C*. *albicans* and *P*. *aeruginosa* were also identified. These organisms have previously been recovered from ET biofilms [1], [4]. Members of the Enterobacteriaceae detected included *E*. *coli*, *E*. *aerogenes* and *K*. *pneumoniae*. Enterobacteriaceae have been isolated from ET biofilms before [1], [3], [4], [13] and the species identified include known respiratory pathogens [38]–[42]. Besides the bacteria frequently isolated from ET biofilms in previous studies, other potential human pathogens were also recovered. These bacteria were only found on a small number of ETs (1 to 3 ETs) and include *S. maltophilia*, *A.lwoffii*, *Pasteurella* spp., *Moraxella* spp., *P*. *damselae*, *M*. *odoratus*, *S*. *paucimobilis* and *K*. *varians* [43]–[53]. In addition, bacteria that have previously not been associated with human infections were found, i.e. *B*. *simplex* and *L*. *aquatica*. Whether these species present a clinical risk remains to be determined.

Four patients were extubated and subsequently re-intubated in the course of our study. Both ETs were investigated and overall there was little similarity between both ETs. Whether this is due to biological variation and/or inherent limitation of our sampling strategy is at present unclear.

### Comparison of Culture Dependent Identification Results and Analyses of the Clone Libraries

Good’s coverage coefficient and collector’s curves showed that the data obtained by sequencing of the clone libraries reflected the bacterial diversity in an accurate way. On the other hand, the coverage obtained by cultivation was highly variable (ranging from 0 to 96). The number of species per ET biofilm found by analyzing the clone libraries and by cultivation was similar (on average 3 species were found per sample). In addition, the Simpson diversity index did not differ significantly between both methods (p>0.05).

Enterobacteriaceae were the most frequently recovered group from the clone libraries. The diversity of Enterobacteriaceae observed by culture-based analysis was higher than the diversity observed in the clone libraries. Lactic acid bacteria were also encountered in the clone libraries and included *G*. *haemolysans*, *S*. *pneumoniae*, *L*. *fermentum* and *E*. *faecium*; however, these species were rarely isolated. Generally, lactic acid bacteria are important members of the human oral flora [54]–[58] but they can also cause infections such as caries and pneumonia [54], [55]. In addition, lactic acid bacteria such as enterococci have been linked to the development of late-onset VAP [23]. Analysis of the clone libraries confirmed the results of the culture-based approach for the presence of *P*. *aeruginosa*, *M*. *luteus*, *S*. *aureus* and *S*. *epidermidis*. For example, *P*. *aeruginosa* was identified on 3 ETs by sequencing the clone libraries and by cultivation; analyzing the clone libraries showed the presence of *P*. *aeruginosa* in 2 additional samples. However, in general, the results obtained by sequencing the clone libraries and by cultivation were very different and, overall there was little correlation between the results of both methods, indicating that a combined approach is needed to obtain a complete picture of the bacterial diversity of ET biofilms.

### Additional Diversity Revealed by Pyrosquencing

Four samples were also analyzed by pyrosequencing. Cultivation and sequencing the clone library revealed that sample E1 was dominated by Enterobacteriaceae and pyrosequencing confirmed the presence of *E*. *aerogenes*, *E*. *coli* and *R*. *ornithinolytica*. In addition, pyrosequencing showed that the diversity among the Enterobacteriaceae was higher than detected by the other methods. This was not surprising as pyrosequencing is a powerful method which allows much deeper sequencing than traditional methods [55], [58], [59]. *In silico* analysis assigned nearly 29000 reads to the Enterobacteriaceae but identification to the species level failed. This is due to the limited resolution of 16 S rRNA gene sequencing for the identification of Enterobacteriaceae. Besides representatives of the Enterobacteriaceae in this sample, only *M*. *salivarium* was detected by pyrosequencing. *M. salivarium* is a member of the normal oral flora [60] and as ET biofilms are partially inoculated by oropharyngeal secretions [19], it is not surprising that *M*. *salivarium* was isolated from ET samples. Pyrosequencing of sample E4 revealed a high bacterial diversity. In contrast, cultivation revealed only a low diversity as a limited number of species were detected, i.e. *E*. *aerogenes*, *E*. *coli*, *Staphylococcus cohnii*, *S*. *epidermidis* and *S*. *xylosus*; the same low diversity was found upon analysis of the clone library. It has already been reported that biofilm formation on the ET is due to aspiration and accumulation of oral secretions [61], [62]. Perkins *et al.* [19] found that over 70% of 16 S rRNA gene sequences in the ET biofilm were associated with genera of the typical oral flora. So, while it is not surprising that we found a high number of representatives of the normal oral flora in the ET biofilm, it is unexpected that representatives of the normal oral flora were not identified among the clones. Pyrosequencing of sample E13 revealed the presence of Actinobacteria, Bacteroidetes, Clostridia, lactic acid bacteria and *P*. *aeruginosa*. The diversity in sample E13 was higher than observed by cultivation or by analysis of the clone library. Pyrosequencing of the sample E17 again revealed a high diversity and representatives of the same groups as detected in sample E4 and E13 were identified, including Actinobacteria, Bacteroidetes, Fusobacteria, γ-Proteobacteria and lactic acid bacteria. Both culture dependent identification and sequencing of the clone library confirmed the presence of *P*. *aeruginosa* but failed to detect the other taxa.

In general, pyrosequencing revealed a higher bacterial diversity than the other methods; the Simpson diversity indexes for the 4 samples studied by pyrosequencing was significantly higher (p<0.05) than those for cultivation and clone libraries. Although members of the normal oral flora constituted a considerable fraction of the pyrosequences, they were not encountered by sequencing the 16 S rRNA gene clone libraries, again highlighting that pyrosequencing allows a more comprehensive analysis of biodiversity [59], [63]. Culture dependent analysis and sequencing of the clone libraries revealed fewer bacterial species but led to the identification of the clinically most relevant species. Differences between the culture dependent identification results and culture independent identification results can be explained in several ways. First of all, the cultivation methods that we used were not suited for the isolation of all bacteria. For example, some members of the normal oral flora are fastidious anaerobic bacteria [55] and would not be isolated using the growth conditions of the present study. Secondly, some bacteria may constitute only a very minor fraction of the biofilm flora and would go unnoticed by sequencing the 16 S rRNA gene clone libraries. However, the use of selective growth conditions may allow the recovery of these bacteria. For instance, *Staphylococcus* spp. were rarely detected by sequencing of clone libraries and by pyrosequencing while cultivation showed their predominance. It is possible that *Staphylococcus* spp. only constituted a small fraction of the ET biofilm flora but that the use of MSA and BPA plates promoted their growth. Also, the use of a standard lysis buffer in order to extract DNA can result in a lower detection of staphylococci by culture independent techniques [64]. A remaining question is whether the oral flora significantly contributes to the pathogenesis of VAP. Although some members of the oral flora are well-known opportunistic pathogens, most of them are low-virulence bacteria. It is possible that members of the normal oral flora form a biofilm on the ET, providing a suitable environment for the growth of late-onset VAP pathogens such as *S*. *aureus* and *P*. *aeruginosa*. Interactions between *C. albicans*, a common colonizer of the oral cavity, and *P. aeruginosa* and *S*. *aureus* are well known [23]. Some of these interactions significantly contribute to pathogenicity, e.g. colonization of the respiratory tract with *C*. *albicans* is associated with an increased risk of *P*. *aeruginosa* VAP [65].

However, repeated sampling of the ET biofilm and more clinical data are necessary to elucidate the mechanism of colonization of the ET.

### Linking Microbiological Data to Clinical Outcome

We analyzed 55 samples by culture dependent identification techniques; 14 of these originated from patients with VAP. To investigate whether the bacterial flora of ET biofilms recovered from patients with or without VAP was different, we compared the occurrence of the most relevant pathogenic groups (CoNS, Enterobacteriaceae, *P*. *aeruginosa*, *S*. *aureus* and *S*. *epidermidis*). The presence of these bacterial groups did not significantly differ between both groups of patients (p>0.05 for all comparisons): CoNS (18/41 non-VAP ETs versus 7/14 VAP ETs), Enterobacteriaceae (11/41 versus 3/14), *P*. *aeruginosa* (4/41 versus 4/14), *S*. *aureus* (10/41 versus 1/14) and *S*. *epidermidis* (25/41 versus 8/14).

We found no significant differences between the microbial flora in ET biofilms (as determined by cultivation) from patients who had previously developed pneumonia and those who did not. In addition, when we compared the microbial flora revealed by culture independent analyses and the occurrence of VAP, we were not able to find any correlation. Also, the results of the Unifrac analysis (performed on 16 S rRNA gene sequences of clones and isolates) demonstrated that there were no differences between the bacterial populations of patients with and without VAP (p = 0.66) or between patients who were intubated more than 5 days and those which were intubated less than 5 days (p = 0.51).

However, the relatively small sample size and the presence of confounding factors (including antimicrobial therapy and underlying condition) may partially explain this apparent lack of correlation that is in contrast with the results of some previous studies. For example, Adair *et al.* [4] found a link between pathogens present in ET biofilms and those isolated from infected lungs in 70% ( = 14/20) of VAP patients. It should be noted, however, that the ET biofilm analysis in this study (as well in ours) represented a cumulative net state at the time of extubation. Thus, the presence of pathogens in the ET biofilm may as well be secondary to the proliferation of pathogens in the distal airways of patients developing VAP. Only a study design in which the ET biofilm is sampled and cultured repetitively, starting immediately following intubation, may disclose a causal relationship between ET biofilm formation and VAP. However, repeated sampling of the ET biofilm is often not allowed due to concerns for patient safety.

### Conclusion

We analyzed ET biofilms by means of culture dependent and independent techniques. The bacterial diversity of the biofilm flora was high and a wide variety of phylogenetic groups was detected. For most samples, cultivation resulted in the isolation of the clinically most relevant groups (*S*. *aureus*, *S*. *epidermidis* and *P*. *aeruginosa*). However, for some samples in which these organisms were not detected by culture-based methods, 16 S rRNA gene clone libraries revealed their presence, again highlighting the added value of culture independent approaches. In contrast, pyrosequencing analysis mostly detected members of the normal oral flora such as *Prevotella* spp. and lactic acid bacteria. A combination of different methods would be necessary to obtain a complete picture of the bacterial diversity of ET biofilms.

## Supporting Information

Figure S1 Collector’s curves of the pyrosequencing experiments. The cut-off for species delineation was 99% sequence similarity.
(TIF)

Table S1 Results of the culture dependent identification techniques (lac: lactose; glu: glucose; CA: cetrimide agar; MSA: mannitol salt agar; BPA: Baird-Parker agar; MHA: Mueller-Hinton agar; VRBGA: violet red bile glucose agar).
(DOCX)

Table S2 Identification results of the 16 S rRNA gene sequencing. The numbers of sequences per species are also given.
(DOCX)

Table S3 Simpson index of diversity (1-D) and Good’s coverage coefficients (C) of ET biofilms investigated by cultivation (cult), 16 S rRNA gene clone libraries (cl) and pyrosequencing of PCR amplified 16 S rRNA genes (pyro).
(DOCX)

Table S4 Collector’s data of the first five clone libraries (cE1 to cE5). The cut-off for species delineation was 99% sequence similarity. OTU: operational taxonomic unit.
(DOCX)
