# Supplementary material for: Assessment of Microbial Diversity in Biofilms Recovered from Endotracheal Tubes Using Culture Dependent and Independent Approaches
Source: PLoS One. 2012 Jun 5;7(6):e38401. doi: 10.1371/journal.pone.0038401 (PMC3367921; doi:10.1371/journal.pone.0038401)
Supplement: Table S1 — Results of the culture dependent identification techniques (lac: lactose; glu: glucose; CA: cetrimide agar; MSA: mannitol salt agar; BPA: Baird-Parker agar; MHA: Mueller-Hinton agar; VRBGA: violet red bile glucose agar). (DOCX) [file pone.0038401.s002.docx]

| **Isolate** | **Medium** | **Sample** | **Gram-stain** | **Cell shape** | **Oxidase** | **Catalase** | **Kligler** | **Growth at 42°C** | **Growth on CA** | **Coagulase** | **DNAse** | **Identification** |
| --- | --- | --- | --- | --- | --- | --- | --- | --- | --- | --- | --- | --- |
| ET-001 | VRBGA | E1 | - | rod | - | + | lac+/glu+ |  |  |  |  | *Enterobacter aerogenes* |
| ET-002 | VRBGA | E1 | - | rod | - | + | lac+/glu+ |  |  |  |  | *Enterobacter aerogenes* |
| ET-003 | VRBGA | E1 | - | rod | - | + | lac+/glu+ |  |  |  |  | *Enterobacter aerogenes* |
| ET-004 | BPA | E1 | + | coccus |  | + |  |  |  | - | - | *Staphylococcus lentus* |
| ET-005 | BPA | E1 | + | coccus |  | + |  |  |  | - | - | *Staphylococcus capitis* |
| ET-006 | MHA | E1 | - | rod | - | + | lac+/glu+ |  |  |  |  | *Enterobacter aerogenes* |
| ET-007 | MHA | E1 | - | rod | - |  | lac+/glu+ |  |  |  |  | *Raoultella ornithinolytica* |
| ET-008 | MHA | E1 | yeast | yeast |  |  |  |  |  |  |  | *Candida albicans* |
| ET-009 | MHA | E1 | - | rod | - | + | lac+/glu+ |  |  |  |  | *Raoultella ornithinolytica* |
| ET-010 | MHA | E1 | - | rod | - | + | lac-/glu+ |  |  |  |  | *Raoultella ornithinolytica* |
| ET-011 | MHA | E1 | - | rod | - | + | lac+/glu+ |  |  |  |  | *Enterobacter aerogenes* |
| ET-012 | MHA | E1 | - | rod | - | + | lac+/glu+ |  |  |  |  | *Escherichia coli* |
| ET-013 | MHA | E1 | - | rod | - | + | lac-/glu+ |  |  |  |  | *Raoultella ornithinolytica* |
| ET-014 | MHA | E2 | + | coccus |  | + |  |  |  | - | - | *Staphylococcus epidermidis* |
| ET-015 | MSA | E2 | + | coccus |  | + |  |  |  | - | - | *Staphylococcus epidermidis* |
| ET-016 | MSA | E2 | - | rod | - | + | lac+/glu+ |  |  |  |  | *Enterobacter aerogenes* |
| ET-017 | BPA | E3 | + | coccus |  | + |  |  |  | - | - | *Staphylococcus epidermidis* |
| ET-018 | MHA | E3 | - | rod | - |  | lac+/glu+ |  |  |  |  | *Raoultella ornithinolytica* |
| ET-019 | BPA | E3 | + | coccus |  | + |  |  |  | - | - | *Staphylococcus xylosus* |
| ET-020 | MHA | E3 | + | coccus |  | + |  |  |  | - | - | *Staphylococcus warneri* |
| ET-021 | BPA | E2 | + | coccus |  | + |  |  |  | - | - | *Staphylococcus epidermidis* |
| ET-022 | MHA | E1 | - | rod | - | + | lac+/glu+ |  |  |  |  | *Raoultella ornithinolytica* |
| ET-023 | MHA | E1 | yeast | yeast |  |  |  |  |  |  |  | *Candida albicans* |
| ET-024 | MHA | E1 | yeast | yeast |  |  |  |  |  |  |  | *Candida albicans* |
| ET-025 | BPA | E4 | + | coccus |  | + |  |  |  | - | - | *Staphylococcus epidermidis* |
| ET-026 | MHA | E4 | - | rod | - | + | lac+/glu+ |  |  |  |  | *Escherichia coli* |
| ET-027 | MHA | E4 | - | rod | - | + | lac+/glu+ |  |  |  |  | *Enterobacter aerogenes* |
| ET-028 | MHA | E4 | + | coccus |  | + |  |  |  | - | - | *Staphylococcus cohnii* |
| ET-029 | MHA | E4 | - | rod | - | + | lac+/glu+ |  |  |  |  | *Escherichia coli* |
| ET-030 | MHA | E4 | + | coccus |  | + |  |  |  | - | - | *Staphylococcus epidermidis* |
| ET-031 | VRBGA | E4 | - | rod | - | + | lac+/glu+ |  |  |  |  | *Escherichia coli* |
| ET-032 | VRBGA | E4 | - | rod | - | + | lac+/glu+ |  |  |  |  | *Enterobacter aerogenes* |
| **Isolate** | **Medium** | **Sample** | **Gram-stain** | **Cell shape** | **Oxidase** | **Catalase** | **Kligler** | **Growth at 42°C** | **Growth on CA** | **Coagulase** | **DNAse** | **Identification** |
| ET-033 | MHA | E4 | - | rod | - | + | lac+/glu+ |  |  |  |  | *Escherichia coli* |
| ET-034 | MHA | E4 | - | rod | - | + | lac+/glu+ |  |  |  |  | *Enterobacter aerogenes* |
| ET-035 | MHA | E4 | - | rod | - | + | lac+/glu+ |  |  |  |  | *Escherichia coli* |
| ET-036 | MSA | E4 | yeast | yeast |  |  |  |  |  |  |  | *Candida spp.* |
| ET-037 | MSA | E4 | + | coccus | - | + |  |  |  | - | - | *Staphylococcus xylosus* |
| ET-038 | MSA | E4 | + | coccus | - | + |  |  |  | - | - | *Staphylococcus xylosus* |
| ET-039 | BPA | E4 | + | coccus | - | + |  |  |  | - | - | *Staphylococcus epidermidis* |
| ET-040 | MHA | E5 | - | rod | - |  | lac+/glu+ |  |  |  |  | *Escherichia coli* |
| ET-041 | MHA | E5 | + | coccus |  | + |  |  |  | - | - | *Staphylococcus warneri* |
| ET-042 | MHA | E5 | yeast | yeast |  |  |  |  |  |  |  | *Candida spp.* |
| ET-043 | BPA | E5 | + | coccus |  | + |  |  |  | - | - | *Staphylococcus epidermidis* |
| ET-044 | MSA | E5 | + | coccus |  | + |  |  |  | + | + | *Staphylococcus aureus* |
| ET-045 | MHA | E5 | + | coccus |  | + |  |  |  | - | - | *Staphylococcus warneri* |
| ET-046 | MHA | E5 | + | coccus |  | - |  |  |  | - | - | *Enterococcus faecium* |
| ET-047 | MSA | E5 | + | coccus |  | + |  |  |  | - | - | *Staphylococcus epidermidis* |
| ET-048 | MSA | E5 | + | coccus |  | + |  |  |  | - | - | *Staphylococcus epidermidis* |
| ET-049 | MHA | E5 | + | coccus |  | + |  |  |  | - | - | *Staphylococcus epidermidis* |
| ET-050 | BPA | E5 | + | coccus |  | + |  |  |  | - | - | *Staphylococcus epidermidis* |
| ET-051 | VRBGA | E5 | - | rod | - |  | lac-/glu+ |  |  |  |  | *Shigella spp.* |
| ET-052 | VRBGA | E5 | - | rod | - |  |  |  |  |  |  | *Acinetobacter lwoffi* |
| ET-053 | BPA | E6 | + | coccus |  | + |  |  |  | - | - | *Staphylococcus epidermidis* |
| ET-054 | MSA | E6 | + | coccus |  | + |  |  |  | - | - | *Staphylococcus epidermidis* |
| ET-055 | MSA | E6 | + | coccus |  | + |  |  |  | - | - | *Staphylococcus hyicus* |
| ET-056 | MHA | E7 | + | coccus |  | + |  |  |  | - | - | *Staphylococcus pasteurii* |
| ET-057 | MHA | E7 | + | coccus |  | + |  |  |  | - | - | *Staphylococcus hominis* |
| ET-058 | BPA | E7 | + | coccus |  | + |  |  |  | + | + | *Staphylococcus aureus* |
| ET-059 | MHA | E7 | + | coccus |  | + |  |  |  | + | + | *Staphylococcus aureus* |
| ET-060 | MSA | E7 | + | coccus |  | + |  |  |  | - | - | *Staphylococcus epidermidis* |
| ET-061 | MHA | E8 | + | coccus |  | + |  |  |  | - | - | *Staphylococcus epidermidis* |
| ET-062 | MHA | E8 | - | rod | - | + |  |  |  |  |  | *Photobacterium damselae* |
| ET-063 | MHA | E8 | - | rod | - | + |  |  |  |  |  | *Pasteurella spp.* |
| ET-064 | MHA | E9 | - | rod | + | + |  |  |  |  |  | *Moraxella spp.* |
| ET-065 | MHA | E9 | - | rod | - |  |  |  |  |  |  | *Photobacterium damselae* |
| **Isolate** | **Medium** | **Sample** | **Gram-stain** | **Cell shape** | **Oxidase** | **Catalase** | **Kligler** | **Growth at 42°C** | **Growth on CA** | **Coagulase** | **DNAse** | **Identification** |
| ET-066 | MHA | E9 | - | coccus |  |  |  |  |  |  |  | *Myroides odoratus* |
| ET-067 | MHA | E10 | + | coccus |  | + |  |  |  | - | - | *Staphylococcus epidermidis* |
| ET-068 | MSA | E10 | + | coccus |  | + |  |  |  | - | - | *Micrococcus luteus* |
| ET-069 | BPA | E10 | + | coccus |  | - |  |  |  |  |  | *Lactococcus lactis* |
| ET-070 | MHA | E10 | yeast | yeast |  |  |  |  |  |  |  | *Candida albicans* |
| ET-071 | BPA | E10 | + | coccus |  | + |  |  |  | - | - | *Staphylococcus haemolyticus* |
| ET-072 | MHA | E10 | yeast | yeast |  |  |  |  |  |  |  | *Candida albicans* |
| ET-073 | MHA | E11 | + | coccus |  | + |  |  |  | - | - | *Staphylococcus epidermidis* |
| ET-074 | MHA | E11 | + | coccus |  | + |  |  |  | - | - | *Staphylococcus capitis* |
| ET-075 | BPA | E11 | + | coccus |  | + |  |  |  | - | - | *Staphylococcus epidermidis* |
| ET-076 | MSA | E11 | + | coccus |  | + |  |  |  | - | - | *Staphylococcus epidermidis* |
| ET-077 | MSA | E11 | + | coccus |  | + |  |  |  | - | - | *Staphylococcus epidermidis* |
| ET-078 | MSA | E11 | + | coccus |  | + |  |  |  | - | - | *Micrococcus luteus* |
| ET-079 | MSA | E11 | + | coccus |  | + |  |  |  | - | - | *Staphylococcus haemolyticus* |
| ET-080 | MHA | E11 | + | coccus |  | + |  |  |  | - | - | *Staphylococcus epidermidis* |
| ET-081 | VRBGA | E12 | - | rod | + |  |  | + | + |  |  | *Pseudomonas aeruginosa* |
| ET-082 | MHA | E12 | + | coccus |  | - |  |  |  |  |  | *Enterococcus faecium* |
| ET-083 | MHA | E12 | + | coccus |  | + |  |  |  | - | - | *Staphylococcus epidermidis* |
| ET-084 | MSA | E12 | + | coccus |  | + |  |  |  | - | - | *Staphylococcus hominis* |
| ET-085 | MSA | E12 | + | coccus |  | + |  |  |  | - | - | *Staphylococcus epidermidis* |
| ET-086 | BPA | E12 | + | coccus |  | - |  |  |  |  |  | *Enterococcus faecium* |
| ET-087 | MHA | E12 | - | rod | + | - |  |  |  |  |  | *Sphingomonas paucimobilis* |
| ET-088 | MHA | E12 | + | coccus |  | + |  |  |  |  |  | *Kocuria varians* |
| ET-089 | MHA | E12 | + | coccus |  | + |  |  |  | - | - | *Staphylococcus hominis* |
| ET-090 | MHA | E12 | + | coccus |  | + |  |  |  | - | - | *Staphylococcus epidermidis* |
| ET-091 | MHA | E12 | + | coccus |  | + |  |  |  | - | - | *Micrococcus luteus* |
| ET-092 | BPA | E12 | + | coccus |  |  |  |  |  | - | - | *Staphylococcus xylosus* |
| ET-093 | MSA | E12 | + | coccus |  | + |  |  |  | - | - | *Staphylococcus saprophyticus* |
| ET-094 | MHA | E12 | - | rod | + |  |  | + | + |  |  | *Pseudomonas aeruginosa* |
| ET-095 | MSA | E12 | + | coccus |  | + |  |  |  | - | - | *Staphylococcus epidermidis* |
| ET-096 | BPA | E12 | + | coccus |  | + |  |  |  | - | - | *Staphylococcus saprophyticus* |
| ET-097 | MHA | E12 | - | rod | + |  |  | + | + |  |  | *Pseudomonas aeruginosa* |
| ET-098 | MHA | E12 | + | coccus |  | + |  |  |  | - | - | *Micrococcus luteus* |
| **Isolate** | **Medium** | **Sample** | **Gram-stain** | **Cell shape** | **Oxidase** | **Catalase** | **Kligler** | **Growth at 42°C** | **Growth on CA** | **Coagulase** | **DNAse** | **Identification** |
| ET-099 | MSA | E12 | + | coccus |  | + |  |  |  | - | - | *Staphylococcus epidermidis* |
| ET-100 | CA | E13 | - | rod | + |  |  | + | + |  |  | *Pseudomonas aeruginosa* |
| ET-101 | CA | E13 | - | rod | + | + |  | + | + |  |  | *Pseudomonas aeruginosa* |
| ET-102 | MSA | E13 | - | rod | - | + | lac+/glu+ |  |  |  |  | *Escherichia coli* |
| ET-103 | BPA | E13 | + | rod |  | + |  |  |  | - | - | *Staphylococcus capitis* |
| ET-104 | CA | E13 | - | rod | + |  |  | + | + |  |  | *Pseudomonas aeruginosa* |
| ET-105 | BPA | E13 | + | coccus |  | + |  |  |  | + | + | *Staphylococcus aureus* |
| ET-106 | MSA | E13 | + | coccus | + |  |  |  |  | - | - | *Staphylococcus epidermidis* |
| ET-107 | CA | E13 | - | rod | + |  |  | + | + |  |  | *Pseudomonas aeruginosa* |
| ET-108 | CA | E13 | - | rod | + |  |  | + | + |  |  | *Pseudomonas aeruginosa* |
| ET-109 | CA | E13 | - | rod | + |  |  | + | + |  |  | *Pseudomonas aeruginosa* |
| ET-110 | MSA | E13 | + | coccus |  | + |  |  |  | - | - | *Staphylococcus epidermidis* |
| ET-111 | MSA | E13 | - | rod | - | + |  |  |  |  |  | *Acinetobacter lwoffi* |
| ET-112 | CA | E13 | - | rod | + |  |  | + | + |  |  | *Pseudomonas aeruginosa* |
| ET-113 | CA | E13 | - | rod | + |  |  | + | + |  |  | *Pseudomonas aeruginosa* |
| ET-114 | CA | E13 | - | rod | + |  |  | + | + |  |  | *Pseudomonas aeruginosa* |
| ET-115 | CA | E13 | - | rod | + |  |  | + | + |  |  | *Pseudomonas aeruginosa* |
| ET-116 | MHA | E13 | yeast | yeast |  |  |  |  |  |  |  | *Candida spp.* |
| ET-117 | CA | E13 | - | rod | + |  |  | + | + |  |  | *Pseudomonas aeruginosa* |
| ET-118 | MHA | E13 | + | rod | + |  |  |  |  | - | - | *Staphylococcus capitis* |
| ET-119 | CA | E13 | - | rod | + |  |  | + | + |  |  | *Pseudomonas aeruginosa* |
| ET-120 | MHA | E13 | + | rod | - | + |  |  |  |  |  | *Streptomyces spp.* |
| ET-121 | MSA | E14 | + | coccus |  | + |  |  |  | - | - | *Micrococcus luteus* |
| ET-122 | MSA | E14 | + | coccus |  | + |  |  |  | - | - | *Micrococcus luteus* |
| ET-123 | MSA | E14 | + | coccus |  | + |  |  |  | - | - | *Staphylococcus epidermidis* |
| ET-124 | MHA | E14 | - | coccus |  | + |  |  |  |  | - | *Myroides odoratus* |
| ET-125 | MHA | E14 | - | coccus |  |  |  |  |  |  |  | *Myroides odoratus* |
| ET-126 | MHA | E14 | + | coccus |  | + |  |  |  | - | - | *Micrococcus luteus* |
| ET-127 | MSA | E14 | + | coccus |  | + |  |  |  | - | - | *Micrococcus luteus* |
| ET-128 | MSA | E14 | + | coccus |  | + |  |  |  | - | - | *Staphylococcus warneri* |
| ET-129 | MSA | E14 | + | coccus |  | + |  |  |  | - | - | *Staphylococcus epidermidis* |
| ET-130 | BPA | E14 | + | coccus |  | + |  |  |  | + | + | *Staphylococcus aureus* |
| ET-131 | MSA | E14 | + | coccus |  | + |  |  |  | - | - | *Staphylococcus hominis* |
| **Isolate** | **Medium** | **Sample** | **Gram-stain** | **Cell shape** | **Oxidase** | **Catalase** | **Kligler** | **Growth at 42°C** | **Growth on CA** | **Coagulase** | **DNAse** | **Identification** |
| ET-132 | MSA | E14 | + | rod | - |  |  |  |  |  |  | *Bacillus simplex* |
| ET-133 | MHA | E14 | + | rod | - |  |  |  |  |  |  | *Bacillus simplex* |
| ET-134 | MHA | E14 | + | rod | - |  |  |  |  |  |  | *Bacillus simplex* |
| ET-135 | CA | E15 | - | rod | + |  |  | + | + |  |  | *Pseudomonas aeruginosa* |
| ET-136 | MHA | E15 | - | rod | + |  |  | + | + |  |  | *Pseudomonas aeruginosa* |
| ET-137 | CA | E15 | - | rod | + |  |  | + | + |  |  | *Pseudomonas aeruginosa* |
| ET-138 | VRBGA | E15 | - | rod | - |  |  |  |  |  |  | *Photobacterium damselae* |
| ET-139 | CA | E15 | - | rod | + |  |  | + | + |  |  | *Pseudomonas aeruginosa* |
| ET-140 | CA | E15 | - | rod | + |  |  | + | + |  |  | *Pseudomonas aeruginosa* |
| ET-141 | CA | E15 | - | rod | + |  |  | + | + |  |  | *Pseudomonas aeruginosa* |
| ET-142 | MHA | E15 | + | coccus |  | + |  |  |  | - | - | *Micrococcus luteus* |
| ET-143 | CA | E15 | - | rod | + | + |  | + | + |  |  | *Pseudomonas aeruginosa* |
| ET-144 | CA | E15 | - | rod | + | + |  | + | + |  |  | *Pseudomonas aeruginosa* |
| ET-145 | CA | E15 | - | rod | + | + |  | + | + |  |  | *Pseudomonas aeruginosa* |
| ET-146 | CA | E15 | - | rod | + | + |  | + | + |  |  | *Pseudomonas aeruginosa* |
| ET-147 | BPA | E17 | + | coccus |  | + |  |  |  | - | - | *Staphylococcus hominis* |
| ET-148 | BPA | E17 | + | coccus |  | + |  |  |  | - | - | *Staphylococcus epidermidis* |
| ET-149 | MHA | E17 | + | coccus |  | + |  |  |  | - | - | *Micrococcus luteus* |
| ET-150 | MSA | E17 | + | coccus |  | + |  |  |  | - | - | *Staphylococcus cohnii* |
| ET-151 | MHA | E16 | + | rod | - | + |  |  |  |  |  | *Rhodococcus corynebacteroides* |
| ET-152 | BPA | E16 | + | rod |  | + |  |  |  |  |  | *Gemella morbillorum* |
| ET-153 | BPA | E16 | + | coccus |  | + |  |  |  | - | - | *Staphylococcus saprophyticus* |
| ET-154 | BPA | E16 | + | rod |  | + |  |  |  |  |  | *Gemella morbillorum* |
| ET-155 | BPA | E16 | + | coccus |  | + |  |  |  | + | + | *Staphylococcus aureus* |
| ET-156 | BPA | E17 | + | coccus |  | + |  |  |  | - | - | *Micrococcus luteus* |
| ET-157 | MHA | E17 | + | rod | - | + |  |  |  |  |  | *Rhodococcus corynebacteroides* |
| ET-158 | BPA | E17 | + | coccus |  | + |  |  |  |  |  | *Kocuria varians* |
| ET-159 | BPA | E17 | + | coccus |  | + |  |  |  | - | - | *Staphylococcus lentus* |
| ET-160 | MHA | E16 | + | coccus |  | + |  |  |  | - | - | *Micrococcus luteus* |
| ET-161 | MHA | E16 | + | coccus |  | + |  |  |  | - | - | *Micrococcus luteus* |
| ET-162 | MHA | E16 | + | coccus |  | + |  |  |  |  |  | *Kocuria varians* |
| ET-163 | MSA | E16 | + | coccus |  | + |  |  |  | - | - | *Staphylococcus epidermidis* |
| ET-164 | BPA | E16 | + | coccus |  | + |  |  |  | - | - | *Staphylococcus haemolyticus* |
| **Isolate** | **Medium** | **Sample** | **Gram-stain** | **Cell shape** | **Oxidase** | **Catalase** | **Kligler** | **Growth at 42°C** | **Growth on CA** | **Coagulase** | **DNAse** | **Identification** |
| ET-165 | MHA | E16 | + | rod |  | + |  |  |  |  |  | *Leifsonia aquatica* |
| ET-166 | BPA | E16 | + | rod |  | + |  |  |  |  |  | *Gemella morbillorum* |
| ET-167 | BPA | E16 | + | rod |  | + |  |  |  |  |  | *Gemella morbillorum* |
| ET-168 | VRBGA | E16 | + | rod |  | + |  |  |  |  |  | *Gemella morbillorum* |
| ET-169 | BPA | E16 | + | coccus |  | + |  |  |  | + | + | *Staphylococcus aureus* |
| ET-170 | BPA | E16 | + | coccus |  | + |  |  |  | - | - | *Staphylococcus xylosus* |
| ET-171 | CA | E17 | - | rod | + |  |  | + | + |  |  | *Pseudomonas aeruginosa* |
| ET-172 | CA | E17 | - | rod | + |  |  | + | + |  |  | *Pseudomonas aeruginosa* |
| ET-173 | BPA | E17 | - | coccus |  | + |  |  |  | - | - | *Micrococcus luteus* |
| ET-174 | BPA | E17 | + | coccus |  | + |  |  |  | - | - | *Staphylococcus xylosus* |
| ET-175 | BPA | E17 | + | coccus |  | + |  |  |  | - | - | *Staphylococcus haemolyticus* |
| ET-176 | BPA | E16 | + | coccus |  | + |  |  |  | - | - | *Micrococcus luteus* |
| ET-177 | BPA | E16 | + | coccus |  | + |  |  |  | + | + | *Staphylococcus aureus* |
| ET-178 | BPA | E16 | - | rod | + |  |  |  |  |  |  | *Sphingomonas paucimobilis* |
| ET-179 | MHA | E16 | - | rod | + |  |  |  |  |  |  | *Aeromonas sobria* |
| ET-180 | MHA | E17 | + | coccus |  | + |  |  |  | - | - | *Micrococcus luteus* |
| ET-181 | BPA | E17 | + | coccus |  | + |  |  |  | - | - | *Staphylococcus haemolyticus* |
| ET-182 | BPA | E17 | + | coccus |  | + |  |  |  | - | - | *Staphylococcus haemolyticus* |
| ET-183 | MHA | E19 | - | rod | - | + | lac+/glu+ |  |  |  |  | *Enterobacter aerogenes* |
| ET-184 | MHA | E19 | - | rod | - | + | lac+/glu+ |  |  |  |  | *Enterobacter aerogenes* |
| ET-185 | MHA | E19 | + | coccus |  | + |  |  |  | + | + | *Staphylococcus aureus* |
| ET-186 | MHA | E19 | + | coccus |  | + |  |  |  | - | - | *Micrococcus luteus* |
| ET-187 | MHA | E18 | + | coccus |  | + |  |  |  | - | - | *Micrococcus luteus* |
| ET-188 | MHA | E18 | + | coccus |  | + |  |  |  | - | - | *Micrococcus luteus* |
| ET-189 | MHA | E18 | + | coccus |  | + |  |  |  | - | - | *Micrococcus luteus* |
| ET-190 | MSA | E18 | + | coccus |  | + |  |  |  | + | + | *Staphylococcus aureus* |
| ET-191 | MHA | E18 | + | rod | - |  |  |  |  |  |  | *Rhodococcus corynebacteroides* |
| ET-192 | MHA | E18 | + | coccus |  | + |  |  |  | + | + | *Staphylococcus aureus* |
| ET-193 | VRBGA | E18 | - | rod | + |  |  | + | + |  |  | *Pseudomonas aeruginosa* |
| ET-194 | MHA | E18 | yeast | yeast |  |  |  |  |  |  |  | *Candida spp.* |
| ET-195 | VRBGA | E20 | - | rod | - | - | lac-/glu- |  |  |  |  | *Stenotrophomonas maltophilia* |
| ET-196 | VRBGA | E20 | - | rod | - | - | lac-/glu- |  |  |  |  | *Stenotrophomonas maltophilia* |
| ET-197 | MSA | E20 | + | coccus |  | + |  |  |  | - | - | *Staphylococcus haemolyticus* |
| **Isolate** | **Medium** | **Sample** | **Gram-stain** | **Cell shape** | **Oxidase** | **Catalase** | **Kligler** | **Growth at 42°C** | **Growth on CA** | **Coagulase** | **DNAse** | **Identification** |
| ET-198 | MHA | E20 | - | rod | - | - | lac-/glu- |  |  |  |  | *Stenotrophomonas maltophilia* |
| ET-199 | MHA | E20 | - | rod | - | - | lac-/glu- |  |  |  |  | *Stenotrophomonas maltophilia* |
| ET-200 | MHA | E20 | - | rod | - | - | lac-/glu- |  |  |  |  | *Stenotrophomonas maltophilia* |
| ET-201 | MHA | E20 | - | rod | - | - | lac-/glu- |  |  |  |  | *Stenotrophomonas maltophilia* |
| ET-202 | VRBGA | E20 | - | rod | - | - | lac-/glu- |  |  |  |  | *Stenotrophomonas maltophilia* |
| ET-203 | VRBGA | E20 | - | rod | - | - | lac-/glu- |  |  |  |  | *Stenotrophomonas maltophilia* |
| ET-204 | VRBGA | E20 | - | rod | - | - | lac-/glu- |  |  |  |  | *Stenotrophomonas maltophilia* |
| ET-205 | VRBGA | E21 | - | rod | - | + | lac-/glu+ |  |  |  |  | *Enterobacter sakazakii* |
| ET-206 | VRBGA | E21 | - | rod | - | + | lac-/glu+ |  |  |  |  | *Enterobacter sakazakii* |
| ET-207 | VRBGA | E21 | - | rod | - | + | lac-/glu+ |  |  |  |  | *Enterobacter sakazakii* |
| ET-208 | VRBGA | E21 | - | rod | - | + | lac-/glu+ |  |  |  |  | *Enterobacter sakazakii* |
| ET-209 | VRBGA | E21 | - | rod | - | + | lac-/glu+ |  |  |  |  | *Enterobacter sakazakii* |
| ET-210 | VRBGA | E21 | - | rod | - | + | lac-/glu+ |  |  |  |  | *Enterobacter sakazakii* |
| ET-211 | VRBGA | E21 | - | rod | - | + | lac-/glu+ |  |  |  |  | *Enterobacter sakazakii* |
| ET-212 | VRBGA | E21 | - | rod | - | + | lac-/glu+ |  |  |  |  | *Enterobacter sakazakii* |
| ET-213 | VRBGA | E21 | - | rod | - | + | lac-/glu+ |  |  |  |  | *Enterobacter sakazakii* |
| ET-214 | VRBGA | E21 | - | rod | - | + | lac-/glu+ |  |  |  |  | *Enterobacter sakazakii* |
| ET-215 | VRBGA | E21 | - | rod | - | + | lac-/glu+ |  |  |  |  | *Enterobacter sakazakii* |
| ET-216 | VRBGA | E21 | - | rod | - | + | lac-/glu+ |  |  |  |  | *Enterobacter sakazakii* |
| ET-217 | VRBGA | E21 | - | rod | - | + | lac-/glu+ |  |  |  |  | *Enterobacter sakazakii* |
| ET-218 | VRBGA | E21 | - | rod | - | + | lac-/glu+ |  |  |  |  | *Enterobacter sakazakii* |
| ET-219 | VRBGA | E23 | - | rod | + | + |  | + | + |  |  | *Pseudomonas aeruginosa* |
| ET-220 | VRBGA | E23 | - | rod | + | + |  | + | + |  |  | *Pseudomonas aeruginosa* |
| ET-221 | VRBGA | E23 | - | rod | + | + |  | + | + |  |  | *Pseudomonas aeruginosa* |
| ET-222 | VRBGA | E23 | - | rod | + | + |  | + | + |  |  | *Pseudomonas aeruginosa* |
| ET-223 | VRBGA | E23 | - | rod | + | + |  | + | + |  |  | *Pseudomonas aeruginosa* |
| ET-224 | VRBGA | E23 | - | rod | + | + |  | + | + |  |  | *Pseudomonas aeruginosa* |
| ET-225 | VRBGA | E23 | - | rod | + | + |  | + | + |  |  | *Pseudomonas aeruginosa* |
| ET-226 | VRBGA | E23 | - | rod | + | + |  | + | + |  |  | *Pseudomonas aeruginosa* |
| ET-227 | VRBGA | E23 | - | rod | + | + |  | + | + |  |  | *Pseudomonas aeruginosa* |
| ET-228 | VRBGA | E23 | - | rod | + | + |  | + | + |  |  | *Pseudomonas aeruginosa* |
| ET-229 | CA | E23 | - | rod | + | + |  | + | + |  |  | *Pseudomonas aeruginosa* |
| ET-230 | CA | E23 | - | rod | + | + |  | + | + |  |  | *Pseudomonas aeruginosa* |
| **Isolate** | **Medium** | **Sample** | **Gram-stain** | **Cell shape** | **Oxidase** | **Catalase** | **Kligler** | **Growth at 42°C** | **Growth on CA** | **Coagulase** | **DNAse** | **Identification** |
| ET-231 | CA | E23 | - | rod | + | + |  | + | + |  |  | *Pseudomonas aeruginosa* |
| ET-232 | CA | E23 | - | rod | + | + |  | + | + |  |  | *Pseudomonas aeruginosa* |
| ET-233 | CA | E23 | - | rod | + | + |  | + | + |  |  | *Pseudomonas aeruginosa* |
| ET-234 | BPA | E25 | + | coccus | - | + |  |  |  | - | - | *Staphylococcus epidermidis* |
| ET-235 | BPA | E25 | + | coccus | - | + |  |  |  | - | - | *Staphylococcus epidermidis* |
| ET-236 | BPA | E25 | + | coccus | - | + |  |  |  | - | - | *Staphylococcus epidermidis* |
| ET-237 | BPA | E25 | + | coccus | - | + |  |  |  | - | - | *Staphylococcus epidermidis* |
| ET-238 | BPA | E25 | + | coccus | - | + |  |  |  | - | - | *Staphylococcus epidermidis* |
| ET-239 | BPA | E25 | + | coccus | - | + |  |  |  | - | - | *Staphylococcus epidermidis* |
| ET-240 | BPA | E25 | + | coccus | - | + |  |  |  | - | - | *Staphylococcus epidermidis* |
| ET-241 | BPA | E25 | + | coccus | - | + |  |  |  | - | - | *Staphylococcus epidermidis* |
| ET-242 | BPA | E25 | + | coccus | - | + |  |  |  | - | - | *Staphylococcus epidermidis* |
| ET-243 | MSA | E25 | + | coccus | - | + |  |  |  | - | - | *Staphylococcus epidermidis* |
| ET-244 | MSA | E25 | + | coccus | - | + |  |  |  | - | - | *Staphylococcus epidermidis* |
| ET-245 | MSA | E25 | + | coccus | - | + |  |  |  | - | - | *Staphylococcus epidermidis* |
| ET-246 | MSA | E25 | + | coccus | - | + |  |  |  | - | - | *Staphylococcus epidermidis* |
| ET-247 | MSA | E25 | + | coccus | - | + |  |  |  | - | - | *Staphylococcus saprophyticus* |
| ET-248 | MSA | E25 | + | coccus | - | + |  |  |  | - | - | *Staphylococcus epidermidis* |
| ET-249 | MSA | E25 | + | coccus | - | + |  |  |  | - | - | *Staphylococcus epidermidis* |
| ET-250 | MSA | E25 | + | coccus | - | + |  |  |  | - | - | *Staphylococcus epidermidis* |
| ET-251 | VRBGA | E26 | - | rod | - | + | lac+/glu+ |  |  |  |  | *Escherichia coli* |
| ET-252 | VRBGA | E26 | - | rod | - | + | lac+/glu+ |  |  |  |  | *Escherichia coli* |
| ET-253 | BPA | E24 | + | coccus | - | + |  |  |  | - | - | *Micrococcus luteus* |
| ET-254 | BPA | E24 | + | coccus | - | + |  |  |  | - | - | *Micrococcus luteus* |
| ET-255 | MSA | E24 | + | coccus | - | + |  |  |  | - | - | *Staphylococcus saprophyticus* |
| ET-256 | MSA | E24 | + | coccus | - | + |  |  |  | - | - | *Staphylococcus saprophyticus* |
| ET-257 | MSA | E24 | + | coccus | - | + |  |  |  | - | - | *Staphylococcus epidermidis* |
| ET-258 | MSA | E24 | + | coccus | - | + |  |  |  | - | - | *Staphylococcus epidermidis* |
| ET-259 | MSA | E24 | + | coccus | - | + |  |  |  | + | + | *Staphylococcus aureus* |
| ET-260 | MSA | E24 | + | rod | + | + |  |  |  |  |  | *Bacillus simplex* |
| ET-261 | MSA | E24 | + | coccus | - | + |  |  |  | - | - | *Staphylococcus epidermidis* |
| ET-262 | MSA | E24 | + | coccus | - | + |  |  |  | - | - | *Micrococcus luteus* |
| ET-263 | MSA | E24 | + | coccus | - | + |  |  |  | - | - | *Micrococcus luteus* |
| **Isolate** | **Medium** | **Sample** | **Gram-stain** | **Cell shape** | **Oxidase** | **Catalase** | **Kligler** | **Growth at 42°C** | **Growth on CA** | **Coagulase** | **DNAse** | **Identification** |
| ET-264 | VRBGA | E25 | yeast | yeast |  |  |  |  |  |  |  | *Candida albicans* |
| ET-265 | VRBGA | E25 | yeast | yeast |  |  |  |  |  |  |  | *Candida spp.* |
| ET-266 | MSA | E25 | + | coccus | - | + |  |  |  | - | - | *Staphylococcus epidermidis* |
| ET-267 | BPA | E26 | + | coccus | - | + |  |  |  | - | - | *Staphylococcus saprophyticus* |
| ET-268 | MSA | E26 | + | coccus | - | + |  |  |  | - | - | *Staphylococcus epidermidis* |
| ET-269 | BPA | E27 | yeast | yeast |  |  |  |  |  |  |  | *Candida albicans* |
| ET-270 | BPA | E27 | yeast | yeast |  |  |  |  |  |  |  | *Candida albicans* |
| ET-271 | BPA | E27 | yeast | yeast |  |  |  |  |  |  |  | *Candida albicans* |
| ET-272 | MSA | E28 | + | coccus | - | + |  |  |  | + | + | *Staphylococcus aureus* |
| ET-273 | MSA | E28 | + | coccus | - | + |  |  |  | + | + | *Staphylococcus aureus* |
| ET-274 | MSA | E28 | + | coccus | - | + |  |  |  | + | + | *Staphylococcus aureus* |
| ET-275 | MSA | E28 | + | coccus | - | + |  |  |  | + | + | *Staphylococcus aureus* |
| ET-276 | MSA | E28 | + | coccus | - | + |  |  |  | + | + | *Staphylococcus aureus* |
| ET-277 | MSA | E28 | + | coccus | - | + |  |  |  | + | + | *Staphylococcus aureus* |
| ET-278 | MSA | E28 | + | coccus | - | + |  |  |  | + | + | *Staphylococcus aureus* |
| ET-279 | MSA | E28 | + | coccus | - | + |  |  |  | + | + | *Staphylococcus aureus* |
| ET-280 | MSA | E28 | + | coccus | - | + |  |  |  | + | + | *Staphylococcus aureus* |
| ET-281 | MSA | E28 | + | coccus | - | + |  |  |  | + | + | *Staphylococcus aureus* |
| ET-282 | MSA | E28 | + | coccus | - | + |  |  |  | + | + | *Staphylococcus aureus* |
| ET-283 | MSA | E28 | + | coccus | - | + |  |  |  | + | + | *Staphylococcus aureus* |
| ET-284 | MSA | E28 | + | coccus | - | + |  |  |  | + | + | *Staphylococcus aureus* |
| ET-285 | MSA | E28 | + | coccus | - | + |  |  |  | + | + | *Staphylococcus aureus* |
| ET-286 | BPA | E28 | + | coccus | - | + |  |  |  | + | + | *Staphylococcus aureus* |
| ET-287 | BPA | E28 | + | coccus | - | + |  |  |  | + | + | *Staphylococcus aureus* |
| ET-288 | BPA | E28 | + | coccus | - | + |  |  |  | + | + | *Staphylococcus aureus* |
| ET-289 | BPA | E28 | + | coccus | - | + |  |  |  | + | + | *Staphylococcus aureus* |
| ET-290 | BPA | E28 | + | coccus | - | + |  |  |  | + | + | *Staphylococcus aureus* |
| ET-291 | BPA | E28 | + | coccus | - | + |  |  |  | + | + | *Staphylococcus aureus* |
| ET-292 | BPA | E28 | + | coccus | - | + |  |  |  | + | + | *Staphylococcus aureus* |
| ET-293 | BPA | E28 | + | coccus | - | + |  |  |  | + | + | *Staphylococcus aureus* |
| ET-294 | BPA | E28 | + | coccus | - | + |  |  |  | + | + | *Staphylococcus aureus* |
| ET-295 | BPA | E28 | + | coccus | - | + |  |  |  | + | + | *Staphylococcus aureus* |
| ET-296 | BPA | E28 | + | coccus | - | + |  |  |  | + | + | *Staphylococcus aureus* |
| **Isolate** | **Medium** | **Sample** | **Gram-stain** | **Cell shape** | **Oxidase** | **Catalase** | **Kligler** | **Growth at 42°C** | **Growth on CA** | **Coagulase** | **DNAse** | **Identification** |
| ET-297 | BPA | E28 | + | coccus | - | + |  |  |  | + | + | *Staphylococcus aureus* |
| ET-298 | BPA | E28 | + | coccus | - | + |  |  |  | + | + | *Staphylococcus aureus* |
| ET-299 | BPA | E28 | + | coccus | - | + |  |  |  | + | + | *Staphylococcus aureus* |
| ET-300 | VRBGA | E29 | - | rod | - | + | lac+/glu+ |  |  |  |  | *Escherichia coli* |
| ET-301 | VRBGA | E29 | - | rod | - | + | lac+/glu+ |  |  |  |  | *Escherichia coli* |
| ET-302 | MSA | E30 | + | coccus | - | + |  |  |  | - | - | *Staphylococcus saprophyticus* |
| ET-303 | MSA | E30 | + | coccus | - | + |  |  |  | - | - | *Staphylococcus epidermidis* |
| ET-304 | MSA | E30 | + | coccus | - | + |  |  |  | - | - | *Staphylococcus epidermidis* |
| ET-305 | MSA | E30 | + | coccus | - | + |  |  |  | - | - | *Staphylococcus epidermidis* |
| ET-306 | MSA | E30 | + | coccus | - | + |  |  |  | - | - | *Staphylococcus saprophyticus* |
| ET-307 | MSA | E30 | + | coccus | - | + |  |  |  | - | - | *Micrococcus luteus* |
| ET-308 | MSA | E30 | + | coccus | - | + |  |  |  | - | - | *Staphylococcus saprophyticus* |
| ET-309 | MSA | E30 | + | coccus | - | + |  |  |  | - | - | *Staphylococcus epidermidis* |
| ET-310 | MSA | E30 | + | coccus | - | + |  |  |  | - | - | *Staphylococcus epidermidis* |
| ET-311 | MSA | E30 | + | coccus | - | + |  |  |  | - | - | *Micrococcus luteus* |
| ET-312 | MSA | E30 | + | coccus | - | + |  |  |  | - | - | *Staphylococcus epidermidis* |
| ET-313 | BPA | E30 | + | rod | - | - |  |  |  |  |  | *Bacillus simplex* |
| ET-314 | BPA | E30 | + | coccus | - | + |  |  |  | - | - | *Staphylococcus saprophyticus* |
| ET-315 | BPA | E30 | + | coccus | - | + |  |  |  | - | - | *Staphylococcus saprophyticus* |
| ET-316 | BPA | E30 | + | coccus | - | + |  |  |  | - | - | *Staphylococcus epidermidis* |
| ET-317 | BPA | E30 | + | coccus | - | - |  |  |  |  |  | *Enterococcus faecium* |
| ET-318 | BPA | E30 | + | coccus | - | + |  |  |  | - | - | *Staphylococcus saprophyticus* |
| ET-319 | BPA | E30 | + | coccus | - | - |  |  |  |  |  | *Enterococcus faecium* |
| ET-320 | BPA | E30 | + | coccus | - | + |  |  |  | - | - | *Staphylococcus epidermidis* |
| ET-321 | BPA | E30 | + | coccus | - | + |  |  |  | - | - | *Staphylococcus saprophyticus* |
| ET-322 | BPA | E30 | + | coccus | - | - |  |  |  |  |  | *Lactococcus lactis* |
| ET-323 | BPA | E30 | + | coccus | - | + |  |  |  | - | - | *Staphylococcus epidermidis* |
| ET-324 | BPA | E30 | + | coccus | - | + |  |  |  | - | - | *Staphylococcus saprophyticus* |
| ET-325 | BPA | E30 | + | coccus | - | + |  |  |  | - | - | *Staphylococcus saprophyticus* |
| ET-326 | BPA | E30 | + | coccus | - | - |  |  |  |  |  | *Lactococcus lactis* |
| ET-327 | BPA | E31 | + | coccus | - | + |  |  |  | - | - | *Staphylococcus epidermidis* |
| ET-328 | BPA | E31 | + | coccus | - | + |  |  |  | - | - | *Staphylococcus epidermidis* |
| ET-329 | MSA | E32 | + | coccus | - | + |  |  |  | - | - | *Staphylococcus epidermidis* |
| **Isolate** | **Medium** | **Sample** | **Gram-stain** | **Cell shape** | **Oxidase** | **Catalase** | **Kligler** | **Growth at 42°C** | **Growth on CA** | **Coagulase** | **DNAse** | **Identification** |
| ET-330 | MSA | E32 | + | coccus | - | + |  |  |  | - | - | *Staphylococcus epidermidis* |
| ET-331 | MSA | E32 | + | coccus | - | + |  |  |  | - | - | *Staphylococcus epidermidis* |
| ET-332 | MSA | E32 | + | coccus | - | + |  |  |  | - | - | *Staphylococcus epidermidis* |
| ET-333 | MSA | E32 | + | coccus | - | + |  |  |  | - | - | *Staphylococcus epidermidis* |
| ET-334 | MSA | E32 | + | coccus | - | + |  |  |  | - | - | *Staphylococcus epidermidis* |
| ET-335 | BPA | E32 | + | coccus | - | + |  |  |  | - | - | *Staphylococcus epidermidis* |
| ET-336 | BPA | E32 | + | coccus | - | + |  |  |  | - | - | *Staphylococcus epidermidis* |
| ET-337 | BPA | E32 | + | coccus | - | + |  |  |  | - | - | *Staphylococcus epidermidis* |
| ET-338 | BPA | E32 | yeast | yeast |  |  |  |  |  |  |  | *Candida albicans* |
| ET-339 | BPA | E32 | yeast | yeast |  |  |  |  |  |  |  | *Candida albicans* |
| ET-340 | BPA | E32 | + | coccus | - | + |  |  |  | - | - | *Staphylococcus epidermidis* |
| ET-341 | BPA | E32 | + | coccus | - | + |  |  |  | - | - | *Staphylococcus epidermidis* |
| ET-342 | BPA | E32 | + | coccus | - | + |  |  |  | - | - | *Staphylococcus epidermidis* |
| ET-343 | BPA | E32 | yeast | yeast |  |  |  |  |  |  |  | *Candida albicans* |
| ET-344 | VRBGA | E32 | yeast | yeast |  |  |  |  |  |  |  | *Candida albicans* |
| ET-345 | VRBGA | E32 | yeast | yeast |  |  |  |  |  |  |  | *Candida albicans* |
| ET-346 | VRBGA | E32 | yeast | yeast |  |  |  |  |  |  |  | *Candida albicans* |
| ET-347 | VRBGA | E32 | yeast | yeast |  |  |  |  |  |  |  | *Candida albicans* |
| ET-348 | VRBGA | E32 | yeast | yeast |  |  |  |  |  |  |  | *Candida albicans* |
| ET-349 | BPA | E33 | + | coccus | + | + |  |  |  | - | - | *Staphylococcus epidermidis* |
| ET-350 | BPA | E33 | + | coccus | + | + |  |  |  | - | - | *Micrococcus luteus* |
| ET-351 | BPA | E33 | + | coccus | + | + |  |  |  | - | - | *Micrococcus luteus* |
| ET-352 | BPA | E33 | + | coccus | + | + |  |  |  | - | - | *Micrococcus luteus* |
| ET-353 | BPA | E33 | + | coccus | + | + |  |  |  | - | - | *Micrococcus luteus* |
| ET-354 | BPA | E33 | + | coccus | + | + |  |  |  | - | - | *Micrococcus luteus* |
| ET-355 | MSA | E33 | + | coccus | - | + |  |  |  | - | - | *Staphylococcus epidermidis* |
| ET-356 | MSA | E33 | + | coccus | - | + |  |  |  | - | - | *Staphylococcus epidermidis* |
| ET-357 | BPA | E34 | yeast | yeast |  |  |  |  |  |  |  | *Candida albicans* |
| ET-358 | BPA | E34 | yeast | yeast |  |  |  |  |  |  |  | *Candida albicans* |
| ET-359 | BPA | E34 | yeast | yeast |  |  |  |  |  |  |  | *Candida albicans* |
| ET-360 | MHA | E34 | + | coccus | - | + |  |  |  | - | - | *Micrococcus luteus* |
| ET-361 | VRBGA | E35 | yeast | yeast |  |  |  |  |  |  |  | *Candida albicans* |
| ET-362 | VRBGA | E35 | yeast | yeast |  |  |  |  |  |  |  | *Candida albicans* |
| **Isolate** | **Medium** | **Sample** | **Gram-stain** | **Cell shape** | **Oxidase** | **Catalase** | **Kligler** | **Growth at 42°C** | **Growth on CA** | **Coagulase** | **DNAse** | **Identification** |
| ET-363 | VRBGA | E35 | - | rod | - | - | lac-/glu- |  |  |  |  | *Stenotrophomonas maltophilia* |
| ET-364 | BPA | E36 | yeast | yeast |  |  |  |  |  |  |  | *Candida albicans* |
| ET-365 | MSA | E37 | + | coccus | - | + |  |  |  | + | + | *Staphylococcus aureus* |
| ET-366 | MSA | E37 | + | coccus | - | + |  |  |  | + | + | *Staphylococcus aureus* |
| ET-367 | MSA | E37 | + | coccus | - | + |  |  |  | - | - | *Staphylococcus epidermidis* |
| ET-368 | MSA | E37 | + | coccus | - | + |  |  |  | - | - | *Staphylococcus epidermidis* |
| ET-369 | MSA | E37 | + | coccus | - | + |  |  |  | - | - | *Staphylococcus epidermidis* |
| ET-370 | BPA | E37 | + | coccus | - | - |  |  |  |  |  | *Gemella morbillorum* |
| ET-371 | BPA | E37 | + | coccus | - | + |  |  |  | - | - | *Staphylococcus epidermidis* |
| ET-372 | BPA | E37 | + | coccus | - | + |  |  |  | - | - | *Staphylococcus epidermidis* |
| ET-373 | BPA | E37 | + | coccus | - | + |  |  |  | - | - | *Staphylococcus epidermidis* |
| ET-374 | MSA | E37 | + | coccus | - | + |  |  |  | - | - | *Staphylococcus epidermidis* |
| ET-375 | MSA | E37 | + | coccus | - | + |  |  |  | - | - | *Staphylococcus epidermidis* |
| ET-376 | BPA | E37 | + | coccus | - | + |  |  |  | - | - | *Staphylococcus epidermidis* |
| ET-377 | BPA | E37 | + | coccus | - | + |  |  |  | - | - | *Staphylococcus epidermidis* |
| ET-378 | BPA | E37 | + | coccus | - | + |  |  |  | - | - | *Staphylococcus epidermidis* |
| ET-379 | BPA | E38 | + | coccus | - | + |  |  |  | - | + | *Staphylococcus epidermidis* |
| ET-380 | BPA | E39 | + | coccus | - | + |  |  |  | - | + | *Staphylococcus epidermidis* |
| ET-381 | MSA | E39 | + | coccus | - | + |  |  |  | - | + | *Staphylococcus epidermidis* |
| ET-382 | MSA | E39 | + | coccus | - | + |  |  |  | - | + | *Staphylococcus epidermidis* |
| ET-383 | MSA | E39 | + | coccus | - | + |  |  |  | - | + | *Staphylococcus epidermidis* |
| ET-384 | BPA | E39 | + | coccus | - | + |  |  |  | - | + | *Staphylococcus epidermidis* |
| ET-385 | BPA | E40 | + | coccus | - | + |  |  |  | - | + | *Staphylococcus epidermidis* |
| ET-386 | BPA | E40 | + | coccus | - | + |  |  |  | - | + | *Staphylococcus saprophyticus* |
| ET-387 | BPA | E40 | + | coccus | - | + |  |  |  | - | + | *Staphylococcus saprophyticus* |
| ET-388 | BPA | E41 | yeast | yeast |  |  |  |  |  |  |  | *Candida albicans* |
| ET-389 | BPA | E41 | yeast | yeast |  |  |  |  |  |  |  | *Candida albicans* |
| ET-390 | BPA | E41 | + | coccus | - | + |  |  |  | - | - | *Staphylococcus epidermidis* |
| ET-391 | BPA | E41 | + | coccus | - | + |  |  |  | - | - | *Staphylococcus epidermidis* |
| ET-392 | BPA | E41 | + | coccus | - | + |  |  |  | - | - | *Staphylococcus epidermidis* |
| ET-393 | MSA | E41 | yeast | yeast |  |  |  |  |  |  |  | *Candida albicans* |
| ET-394 | MSA | E41 | + | coccus | - | + |  |  |  | - | - | *Staphylococcus epidermidis* |
| ET-395 | MSA | E41 | + | coccus | - | + |  |  |  | - | - | *Staphylococcus epidermidis* |
| **Isolate** | **Medium** | **Sample** | **Gram-stain** | **Cell shape** | **Oxidase** | **Catalase** | **Kligler** | **Growth at 42°C** | **Growth on CA** | **Coagulase** | **DNAse** | **Identification** |
| ET-396 | MSA | E41 | yeast | yeast |  |  |  |  |  |  |  | *Candida albicans* |
| ET-397 | VRBGA | E41 | yeast | yeast |  |  |  |  |  |  |  | *Candida albicans* |
| ET-398 | VRBGA | E41 | yeast | yeast |  |  |  |  |  |  |  | *Candida albicans* |
| ET-399 | VRBGA | E41 | yeast | yeast |  |  |  |  |  |  |  | *Candida albicans* |
| ET-400 | VRBGA | E41 | yeast | yeast |  |  |  |  |  |  |  | *Candida albicans* |
| ET-401 | BPA | E41 | yeast | yeast |  |  |  |  |  |  |  | *Candida albicans* |
| ET-402 | BPA | E41 | yeast | yeast |  |  |  |  |  |  |  | *Candida albicans* |
| ET-403 | BPA | E41 | + | coccus | - | + |  |  |  | - | - | *Staphylococcus epidermidis* |
| ET-404 | MSA | E41 | + | coccus | - | + |  |  |  | - | - | *Staphylococcus epidermidis* |
| ET-405 | VRBGA | E41 | yeast | yeast |  |  |  |  |  |  |  | *Candida albicans* |
| ET-406 | BPA | E41 | + | coccus | - | + |  |  |  | - | - | *Staphylococcus epidermidis* |
| ET-407 | MHA | E42 | + | coccus | - | + |  |  |  | - | - | *Staphylococcus saprophyticus* |
| ET-408 | MHA | E42 | + | coccus | - | + |  |  |  | - | - | *Staphylococcus saprophyticus* |
| ET-409 | MHA | E42 | + | coccus | - | + |  |  |  | + | + | *Staphylococcus aureus* |
| ET-410 | MHA | E42 | + | coccus | - | + |  |  |  | - | - | *Staphylococcus epidermidis* |
| ET-411 | MHA | E42 | + | coccus | - | + |  |  |  | - | - | *Staphylococcus saprophyticus* |
| ET-412 | MHA | E42 | + | coccus | - | + |  |  |  | - | - | *Staphylococcus saprophyticus* |
| ET-413 | MHA | E42 | + | coccus | - | + |  |  |  | - | - | *Staphylococcus saprophyticus* |
| ET-414 | BPA | E43 | + | coccus | - | + |  |  |  | + | + | *Staphylococcus aureus* |
| ET-415 | BPA | E43 | + | coccus | - | + |  |  |  | - | - | *Staphylococcus saprophyticus* |
| ET-416 | BPA | E43 | + | coccus | - | + |  |  |  | + | + | *Staphylococcus aureus* |
| ET-417 | BPA | E43 | + | coccus | - | + |  |  |  | + | + | *Staphylococcus aureus* |
| ET-418 | BPA | E43 | + | coccus | - | + |  |  |  | + | + | *Staphylococcus aureus* |
| ET-419 | BPA | E43 | + | coccus | - | + |  |  |  | + | + | *Staphylococcus aureus* |
| ET-420 | MSA | E43 | + | coccus | - | + |  |  |  | + | + | *Staphylococcus aureus* |
| ET-421 | MSA | E43 | + | coccus | - | + |  |  |  | + | + | *Staphylococcus aureus* |
| ET-422 | MSA | E43 | + | coccus | - | + |  |  |  | + | + | *Staphylococcus aureus* |
| ET-423 | MSA | E43 | + | coccus | - | + |  |  |  | + | + | *Staphylococcus aureus* |
| ET-424 | MSA | E43 | + | coccus | - | + |  |  |  | + | + | *Staphylococcus aureus* |
| ET-425 | MSA | E43 | + | coccus | - | + |  |  |  | + | + | *Staphylococcus aureus* |
| ET-426 | VRBGA | E43 | - | rod | - | + | lac+/glu+ |  |  |  |  | *Klebsiella oxytoca* |
| ET-427 | VRBGA | E43 | - | rod | - | + | lac-/glu+ |  |  |  |  | *Hafnia alvei* |
| ET-428 | BPA | E43 | + | coccus | - | + |  |  |  | + | + | *Staphylococcus aureus* |
| **Isolate** | **Medium** | **Sample** | **Gram-stain** | **Cell shape** | **Oxidase** | **Catalase** | **Kligler** | **Growth at 42°C** | **Growth on CA** | **Coagulase** | **DNAse** | **Identification** |
| ET-429 | BPA | E43 | + | coccus | - | + |  |  |  | + | + | *Staphylococcus aureus* |
| ET-430 | BPA | E43 | + | coccus | - | + |  |  |  | + | + | *Staphylococcus aureus* |
| ET-431 | BPA | E43 | + | coccus | - | + |  |  |  | + | + | *Staphylococcus aureus* |
| ET-432 | BPA | E43 | + | coccus | - | + |  |  |  | + | + | *Staphylococcus aureus* |
| ET-433 | MSA | E43 | + | coccus | - | + |  |  |  | + | + | *Staphylococcus aureus* |
| ET-434 | MSA | E43 | + | coccus | - | + |  |  |  | + | + | *Staphylococcus aureus* |
| ET-435 | MSA | E43 | + | coccus | - | + |  |  |  | + | + | *Staphylococcus aureus* |
| ET-436 | MSA | E43 | + | coccus | - | + |  |  |  | + | + | *Staphylococcus aureus* |
| ET-437 | MSA | E43 | + | coccus | - | + |  |  |  | + | + | *Staphylococcus aureus* |
| ET-438 | BPA | E43 | + | coccus | - | + |  |  |  | + | + | *Staphylococcus aureus* |
| ET-439 | BPA | E43 | + | coccus | - | + |  |  |  | + | + | *Staphylococcus aureus* |
| ET-440 | MSA | E43 | + | coccus | - | + |  |  |  | + | + | *Staphylococcus aureus* |
| ET-441 | MSA | E43 | + | coccus | - | + |  |  |  | + | + | *Staphylococcus aureus* |
| ET-442 | MSA | E43 | + | coccus | - | + |  |  |  | + | + | *Staphylococcus aureus* |
| ET-443 | BPA | E43 | + | coccus | - | + |  |  |  | + | + | *Staphylococcus aureus* |
| ET-444 | BPA | E43 | + | coccus | - | + |  |  |  | + | + | *Staphylococcus aureus* |
| ET-445 | MHA | E44 | + | coccus | + | + |  |  |  | - | - | *Micrococcus luteus* |
| ET-446 | MHA | E44 | - | coccus | + | + |  |  |  |  |  | *Moraxella spp.* |
| ET-447 | MHA | E44 | + | coccus | - | + |  |  |  | - | - | *Staphylococcus epidermidis* |
| ET-448 | MHA | E44 | - | coccus | + | + |  |  |  |  |  | *Moraxella spp.* |
| ET-449 | MHA | E44 | - | coccus | + | + |  |  |  |  |  | *Moraxella spp.* |
| ET-450 | MHA | E44 | + | coccus | - | + |  |  |  | - | - | *Staphylococcus saprophyticus* |
| ET-451 | MHA | E44 | + | coccus | + | + |  |  |  | - | - | *Micrococcus luteus* |
| ET-452 | MHA | E44 | + | coccus | + | + |  |  |  | - | - | *Micrococcus luteus* |
| ET-453 | CA | E45 | - | rod | + | + |  | + | + |  |  | *Pseudomonas aeruginosa* |
| ET-454 | CA | E45 | - | rod | + | + |  | + | + |  |  | *Pseudomonas aeruginosa* |
| ET-455 | VRBGA | E45 | - | rod | + | + |  | + | + |  |  | *Pseudomonas aeruginosa* |
| ET-456 | VRBGA | E45 | - | rod | + | + |  | + | + |  |  | *Pseudomonas aeruginosa* |
| ET-457 | VRBGA | E45 | - | rod | + | + |  | + | + |  |  | *Pseudomonas aeruginosa* |
| ET-458 | CA | E45 | - | rod | + | + |  | + | + |  |  | *Pseudomonas aeruginosa* |
| ET-459 | MSA | E46 | + | rod | - | + |  |  |  |  |  | *Bacillus simplex* |
| ET-460 | MSA | E46 | + | coccus | - | + |  |  |  | - | - | *Micrococcus luteus* |
| ET-461 | MHA | E46 | + | coccus | - | + |  |  |  | - | - | *Staphylococcus saprophyticus* |
| **Isolate** | **Medium** | **Sample** | **Gram-stain** | **Cell shape** | **Oxidase** | **Catalase** | **Kligler** | **Growth at 42°C** | **Growth on CA** | **Coagulase** | **DNAse** | **Identification** |
| ET-462 | MHA | E46 | yeast | yeast |  |  |  |  |  |  |  | *Candida albicans* |
| ET-463 | MHA | E46 | + | rod | - | + |  |  |  |  |  | *Bacillus simplex* |
| ET-464 | MHA | E46 | yeast | yeast |  |  |  |  |  |  |  | *Candida albicans* |
| ET-465 | BPA | E46 | yeast | yeast |  |  |  |  |  |  |  | *Candida albicans* |
| ET-466 | BPA | E46 | yeast | yeast |  |  |  |  |  |  |  | *Candida albicans* |
| ET-467 | VRBGA | E46 | yeast | yeast |  |  |  |  |  |  |  | *Candida albicans* |
| ET-468 | VRBGA | E46 | yeast | yeast |  |  |  |  |  |  |  | *Candida albicans* |
| ET-469 | VRBGA | E46 | yeast | yeast |  |  |  |  |  |  |  | *Candida albicans* |
| ET-470 | VRBGA | E46 | yeast | yeast |  |  |  |  |  |  |  | *Candida albicans* |
| ET-471 | VRBGA | E46 | yeast | yeast |  |  |  |  |  |  |  | *Candida albicans* |
| ET-472 | VRBGA | E46 | yeast | yeast |  |  |  |  |  |  |  | *Candida albicans* |
| ET-473 | BPA | E46 | yeast | yeast |  |  |  |  |  |  |  | *Candida albicans* |
| ET-474 | BPA | E46 | yeast | yeast |  |  |  |  |  |  |  | *Candida albicans* |
| ET-475 | MHA | E46 | yeast | yeast |  |  |  |  |  |  |  | *Candida albicans* |
| ET-476 | MHA | E46 | + | coccus | - | + |  |  |  | - | - | *Micrococcus luteus* |
| ET-477 | MHA | E46 | yeast | yeast |  |  |  |  |  |  |  | *Candida albicans* |
| ET-478 | MHA | E46 | yeast | yeast |  |  |  |  |  |  |  | *Candida albicans* |
| ET-479 | MHA | E46 | + | coccus | - | + |  |  |  | - | - | *Micrococcus luteus* |
| ET-480 | BPA | E46 | yeast | yeast |  |  |  |  |  |  |  | *Candida albicans* |
| ET-481 | VRBGA | E46 | yeast | yeast |  |  |  |  |  |  |  | *Candida albicans* |
| ET-482 | MSA | E47 | + | coccus | - | + |  |  |  | - | - | *Staphylococcus epidermidis* |
| ET-483 | BPA | E47 | + | coccus | - | + |  |  |  | - | - | *Staphylococcus epidermidis* |
| ET-484 | VRBGA | E47 | yeast | yeast |  |  |  |  |  |  |  | *Candida albicans* |
| ET-485 | VRBGA | E47 | yeast | yeast |  |  |  |  |  |  |  | *Candida albicans* |
| ET-486 | VRBGA | E47 | yeast | yeast |  |  |  |  |  |  |  | *Candida albicans* |
| ET-487 | VRBGA | E47 | yeast | yeast |  |  |  |  |  |  |  | *Candida albicans* |
| ET-488 | VRBGA | E49 | - | rod | + | + |  | + | + |  |  | *Pseudomonas aeruginosa* |
| ET-489 | CA | E49 | - | rod | + | + |  | + | + |  |  | *Pseudomonas aeruginosa* |
| ET-490 | CA | E49 | - | rod | + | + |  | + | + |  |  | *Pseudomonas aeruginosa* |
| ET-491 | BPA | E49 | + | coccus | - | + |  |  |  | - | - | *Staphylococcus epidermidis* |
| ET-492 | BPA | E49 | + | coccus | - | + |  |  |  | - | - | *Staphylococcus epidermidis* |
| ET-493 | MSA | E49 | + | coccus | - | + |  |  |  | - | - | *Staphylococcus epidermidis* |
| ET-494 | MSA | E49 | + | coccus | - | + |  |  |  | - | - | *Staphylococcus epidermidis* |
| **Isolate** | **Medium** | **Sample** | **Gram-stain** | **Cell shape** | **Oxidase** | **Catalase** | **Kligler** | **Growth at 42°C** | **Growth on CA** | **Coagulase** | **DNAse** | **Identification** |
| ET-495 | BPA | E49 | + | coccus | - | + |  |  |  | - | - | *Staphylococcus epidermidis* |
| ET-496 | BPA | E49 | + | coccus | - | + |  |  |  | - | - | *Staphylococcus epidermidis* |
| ET-497 | BPA | E48 | - | rod | - | + | lac-/glu+ |  |  |  |  | *Klebsiella pneumoniae* |
| ET-498 | BPA | E48 | - | rod | - | + | lac-/glu+ |  |  |  |  | *Klebsiella pneumoniae* |
| ET-499 | VRBGA | E48 | - | rod | - | + | lac-/glu+ |  |  |  |  | *Klebsiella pneumoniae* |
| ET-500 | VRBGA | E48 | - | rod | - | + | lac-/glu+ |  |  |  |  | *Klebsiella pneumoniae* |
| ET-501 | VRBGA | E48 | - | rod | - | + | lac-/glu+ |  |  |  |  | *Klebsiella pneumoniae* |
| ET-502 | VRBGA | E48 | - | rod | - | + | lac-/glu+ |  |  |  |  | *Klebsiella pneumoniae* |
| ET-503 | VRBGA | E48 | - | rod | - | + | lac-/glu+ |  |  |  |  | *Klebsiella pneumoniae* |
| ET-504 | VRBGA | E48 | - | rod | - | + | lac-/glu+ |  |  |  |  | *Klebsiella pneumoniae* |
| ET-505 | VRBGA | E48 | - | rod | - | + | lac-/glu+ |  |  |  |  | *Klebsiella pneumoniae* |
| ET-506 | VRBGA | E48 | - | rod | - | + | lac-/glu+ |  |  |  |  | *Klebsiella pneumoniae* |
| ET-507 | BPA | E50 | - | rod | - | + | lac-/glu+ |  |  |  |  | *Klebsiella pneumoniae* |
| ET-508 | BPA | E50 | - | rod | - | + | lac-/glu+ |  |  |  |  | *Klebsiella pneumoniae* |
| ET-509 | VRBGA | E50 | - | rod | - | + | lac-/glu+ |  |  |  |  | *Klebsiella pneumoniae* |
| ET-510 | VRBGA | E50 | - | rod | - | + | lac-/glu+ |  |  |  |  | *Klebsiella pneumoniae* |
| ET-511 | BPA | E50 | - | rod | - | + | lac-/glu+ |  |  |  |  | *Klebsiella pneumoniae* |
| ET-512 | BPA | E50 | + | coccus | - | + |  |  |  | - | - | *Micrococcus luteus* |
| ET-513 | VRBGA | E50 | - | rod | - | + | lac-/glu+ |  |  |  |  | *Klebsiella pneumoniae* |
| ET-514 | VRBGA | E50 | - | rod | - | + | lac-/glu+ |  |  |  |  | *Klebsiella pneumoniae* |
| ET-515 | BPA | E50 | - | rod | - | + | lac-/glu+ |  |  |  |  | *Klebsiella pneumoniae* |
| ET-516 | VRBGA | E50 | - | rod | - | + | lac-/glu+ |  |  |  |  | *Klebsiella pneumoniae* |
| ET-517 | VRBGA | E50 | - | rod | - | + | lac-/glu+ |  |  |  |  | *Klebsiella pneumoniae* |
| ET-518 | BPA | E50 | - | rod | - | + | lac-/glu+ |  |  |  |  | *Klebsiella pneumoniae* |
| ET-519 | VRBGA | E50 | - | rod | - | + | lac-/glu+ |  |  |  |  | *Klebsiella pneumoniae* |
| ET-520 | VRBGA | E50 | - | rod | - | + | lac-/glu+ |  |  |  |  | *Klebsiella pneumoniae* |
| ET-521 | VRBGA | E51 | - | rod | - | + | lac-/glu+ |  |  |  |  | *Klebsiella pneumoniae* |
| ET-522 | VRBGA | E51 | - | rod | - | + | lac-/glu+ |  |  |  |  | *Klebsiella pneumoniae* |
| ET-523 | VRBGA | E51 | - | rod | - | + | lac-/glu+ |  |  |  |  | *Klebsiella pneumoniae* |
| ET-524 | VRBGA | E51 | - | rod | - | + | lac-/glu+ |  |  |  |  | *Klebsiella pneumoniae* |
| ET-525 | VRBGA | E51 | - | rod | - | + | lac-/glu+ |  |  |  |  | *Klebsiella pneumoniae* |
| ET-526 | VRBGA | E51 | - | rod | - | + | lac-/glu+ |  |  |  |  | *Klebsiella pneumoniae* |
| ET-527 | VRBGA | E51 | - | rod | - | + | lac-/glu+ |  |  |  |  | *Klebsiella pneumoniae* |
| **Isolate** | **Medium** | **Sample** | **Gram-stain** | **Cell shape** | **Oxidase** | **Catalase** | **Kligler** | **Growth at 42°C** | **Growth on CA** | **Coagulase** | **DNAse** | **Identification** |
| ET-528 | VRBGA | E51 | - | rod | - | + | lac-/glu+ |  |  |  |  | *Klebsiella pneumoniae* |
| ET-529 | MSA | E53 | + | coccus | - | + |  |  |  | - | - | *Staphylococcus saprophyticus* |
| ET-530 | CA | E53 | - | rod | - | + | lac+/glu+ |  |  |  |  | *Enterobacter sakazakii* |
| ET-531 | CA | E53 | - | rod | - | + | lac+/glu+ |  |  |  |  | *Enterobacter sakazakii* |
| ET-532 | BPA | E55 | + | coccus | - | + |  |  |  | - | - | *Staphylococcus epidermidis* |
| ET-533 | MSA | E55 | + | coccus | - | + |  |  |  | - | - | *Staphylococcus epidermidis* |
| ET-534 | MSA | E55 | + | coccus | - | + |  |  |  | - | - | *Staphylococcus epidermidis* |
| ET-535 | MSA | E55 | + | coccus | - | + |  |  |  | - | - | *Staphylococcus epidermidis* |
| ET-536 | MSA | E55 | + | coccus | - | + |  |  |  | - | - | *Micrococcus luteus* |
| ET-537 | BPA | E55 | + | coccus | - | + |  |  |  | - | - | *Staphylococcus epidermidis* |
| ET-538 | BPA | E55 | + | coccus | - | + |  |  |  | - | - | *Staphylococcus epidermidis* |
| ET-539 | MSA | E55 | + | coccus | - | + |  |  |  | - | - | *Staphylococcus epidermidis* |
| ET-540 | MSA | E55 | + | coccus | - | + |  |  |  | - | - | *Staphylococcus epidermidis* |
| ET-541 | MSA | E55 | + | coccus | - | + |  |  |  | - | - | *Staphylococcus epidermidis* |
| ET-542 | MSA | E55 | + | coccus | - | + |  |  |  | - | - | *Staphylococcus epidermidis* |
| ET-543 | BPA | E54 | + | coccus | - | + |  |  |  | - | - | *Staphylococcus epidermidis* |
| ET-544 | BPA | E54 | + | coccus | - | + |  |  |  | - | - | *Staphylococcus epidermidis* |
| ET-545 | BPA | E54 | + | coccus | - | + |  |  |  | - | - | *Staphylococcus epidermidis* |
| ET-546 | MSA | E54 | + | coccus | - | + |  |  |  | - | - | *Staphylococcus epidermidis* |
| ET-547 | MSA | E54 | + | coccus | - | + |  |  |  | - | - | *Staphylococcus saprophyticus* |
| ET-548 | MSA | E54 | + | coccus | - | + |  |  |  | - | - | *Staphylococcus epidermidis* |
| ET-549 | BPA | E54 | + | coccus | - | + |  |  |  | - | - | *Staphylococcus saprophyticus* |
| ET-550 | VRBGA | E54 | yeast | yeast |  |  |  |  |  |  |  | *Candida albicans* |
| ET-551 | VRBGA | E54 | yeast | yeast |  |  |  |  |  |  |  | *Candida albicans* |
| ET-552 | VRBGA | E54 | yeast | yeast |  |  |  |  |  |  |  | *Candida albicans* |
| ET-553 | VRBGA | E54 | yeast | yeast |  |  |  |  |  |  |  | *Candida albicans* |
| ET-554 | VRBGA | E54 | yeast | yeast |  |  |  |  |  |  |  | *Candida albicans* |
