# Supplementary material for: Assessment of Microbial Diversity in Biofilms Recovered from Endotracheal Tubes Using Culture Dependent and Independent Approaches
Source: PLoS One. 2012 Jun 5;7(6):e38401. doi: 10.1371/journal.pone.0038401 (PMC3367921; doi:10.1371/journal.pone.0038401)
Supplement: Table S2 — Identification results of the 16 S rRNA gene sequencing. The numbers of sequences per species are also given. (DOCX) [file pone.0038401.s003.docx]

| *Sample* | Pyrosequencing | | Clone libraries | | 16S rRNA gene sequencing of the isolates | |
| --- | --- | --- | --- | --- | --- | --- |
|  | Bacterial group | Species identification | Bacterial group | Species identification | Bacterial group | Species identification |
| *1* | Enterobacteriaceae | *Enterobacter* *aerogenes* (13323), *Raoultella* *ornithinolytica* (52), *Serratia* *marcescens* (2904), *Enterobacter* *amnigenus* (17), *Cronobacter* *muytjensii* (91), *Klebsiella* *oxytoca* (34), *Ewingella* *americana* (15), *Klebsiella* *pneumoniae* (19), unidentified *Enterobacteriaceae* (28707) | Enterobacteriaceae | *E*. *aerogenes* (10) |  |  |
|  | Actinobacteria | *Mycoplasma salivarium* (3293) | Actinobacteria | *Actinomyces* *odontolyticus* (1) |  |  |
| *2* |  |  | Enterobacteriaceae | *Enterobacter* spp.(6) |  |  |
|  |  |  | Firmicutes | *Staphylococcus* *epidermidis* (6) |  |  |
| *3* |  |  | Enterobacteriaceae | *E*. *aerogenes* (22), *Enterobacter* spp.(4) | Enterobacteriaceae | *R. ornithinolytica* (1) |
| *4* | Actinobacteria | *Atopobium* *parvulum* (206), *Slackia* *exigua* (38) |  |  |  |  |
|  | Bacteroidetes | *Prevotella* *histicola* (2174), *Prevotella melaninogenica* (4084), *Prevotella* *nigrescens* (129), *Prevotella* *oris* (573), *Prevotella* *salivae* (1059) |  |  |  |  |
|  | Clostridia | *Parvimonas* *micra* (21), *Peptostreptococcus* *stomatis* (826), *Selenomonas* *sputigena* (13), *Veillonella* *atypica* (343), *Veillonella* *dispar* (113), *Veillonella* *parvula* (392) |  |  |  |  |
|  | Enterobacteriaceae | *E*. *aerogenes* (18), *Shigella* *boydii* (28), *Enterobacter* *hormaechei* (11), *Escherichia* *coli* (2350), *K*. *oxytoca* (13) |  |  |  |  |
|  | β-Proteobacteria | *Eikenella* *corrodens* (41) |  |  |  |  |
|  | Fusobacteria | *Fusobacterium* *nucleatum* (12), *Sneathia* *sanguinegens* (25) |  |  |  |  |
|  | γ-Proteobacteria | *Aggregatibacter* *segnis* (2040), *Haemophilus* *influenza* (11) |  |  |  |  |
|  | LAB | *Granulicatella* *adiacens* (139), *Streptococcus* *constellatus* (6452), *Streptococcus* *mitis* (325), *Streptococcus* *oralis* (9741), *Streptococcus* *parasanguinis* (128), *Streptococcus* *pneumoniae* (382), *Lactobacillus* *gasseri* (16), *Lactobacillus* *salivarius* (17) | LAB | *Gemella* *haemolysans* (20) |  |  |
|  | Firmicutes | *S.* *epidermidis* (45) | Firmicutes | *Staphylococcus* *aureus* (3) | Firmicutes | *S. epidermidis* (1) |
|  | ε-Proteobacteria | *Campylobacter* *curvus* (15) |  |  |  |  |
| *5* |  |  | Enterobacteriaceae | *E*. *aerogenes* (16), *Klebsiella* spp. (7) |  |  |
|  |  |  |  |  | Firmicutes | *S*. *epidermidis* (1) |
| *6* |  |  | Firmicutes | *S*. *epidermidis* (3) |  |  |
| *7* |  |  | Firmicutes | *S*. *epidermidis* (7) | Firmicutes | *Staphylococcus* *pasteurii* (1), *Staphylococcus* *warneri* (1), *S*. *aureus* (1) |
|  |  |  | Actinobacteria | *Micrococcus* *luteus* (1) |  |  |
| *8* |  |  | Actinobacteria | *M*. *luteus* (4) |  |  |
|  |  |  | Fusobacteria | *Leptotrichia* spp.(4) |  |  |
|  |  |  |  |  | Firmicutes | *S*. *epidermidis* (1) |
| *9* |  |  | Firmicutes | *Bacillus* *cereus* (10), *Staphylococcus* *haemolyticus* (3) |  |  |
|  |  |  | Enterobacteriaceae | *E*. *aerogenes* (8), *Enterobacter* spp. (2) |  |  |
|  |  |  | LAB | *G*. *haemolysans* (17), *Lactobacillus* *fermentum* (12) |  |  |
| *10* |  |  | γ-Proteobacteria | *Pseudomonas* *aeruginosa* (17) |  |  |
|  |  |  | β-Proteobacteria | *Alcaligenes* spp.(2) |  |  |
| *11* |  |  | Actinobacteria | *M*. *luteus* (3) |  |  |
|  |  |  | γ-Proteobacteria | *Photobacterium* spp.(4) |  |  |
| *12* |  |  | LAB | *G*. *haemolysans* (8), *S*. *pneumoniae* (3), *Lactobacillus* *fermentum* (5) | LAB | *Enterococcus* *faecium* (1) |
|  |  |  | Enterobacteriaceae | *E*. *aerogenenes* (7) |  |  |
|  |  |  | Firmicutes | *S*. *epidermidis* (1) | Firmicutes | *S*. *epidermidis* (1) |
|  |  |  | γ-Proteobacteria | *P*. *aeruginosa* (9) |  |  |
|  |  |  |  |  | Actinobacteria | *Kocuria* *rhizophila* (1), *M.* *luteus* (1) |
| *13* | Actinobacteria | *A. odontolyticus* (18), *Actinomyces viscosus* (10), *A.* *parvulum* (24), *Corynebacterium*. *striatum* (283), *Corynebacterium* *propinquum* (87), *Rothia* *mucilaginosa* (647), *Rothia* *dentocariosa* (14) |  |  | Actinobacteria | *Streptomyces* spp. (1) |
|  | Bacteroidetes | *P*. *histicola* (396) |  |  |  |  |
|  | Clostridia | *V*. *parvula* (1106) |  |  |  |  |
|  | LAB | *G.* *adiacens* (4229), *G.* *haemolysans* (1680), *Gemella* *morbillorum* (1455), *Gemella* *sanguinis* (18) |  |  |  |  |
|  | γ-Proteobacteria | *P. aeruginosa* (4675) | γ-Proteobacteria | *P*. *aeruginosa* (11) |  |  |
|  |  |  | Enterobacteriaceae | *Enterobacter* spp. (9) |  |  |
| *14* |  |  | Enterobacteriaceae | *Klebsiella* spp.(8) |  |  |
|  |  |  | γ-Proteobacteria | *P*. *aeruginosa* (13) |  |  |
|  |  |  |  |  | Actinobacteria | *M*. *luteus* (1) |
|  |  |  |  |  | Firmicutes | *S*. *warneri* (1), *Bacillus* *simplex* (3) |
| *15* |  |  | γ-Proteobacteria | *P*. *aeruginosa* (2) |  |  |
|  |  |  | Enterobacteriaceae | *Klebsiella* spp. (4) |  |  |
|  |  |  |  |  | Actinobacteria | *M*. *luteus* (1) |
| *16* |  |  | LAB | *E.* *faecium* (7) |  |  |
|  |  |  | Enterobacteriaceae | *Enterobacter* spp. (5) |  |  |
|  |  |  |  |  | Actinobacteria | *Rhodococcus* *corynebacteroides* (1) |
| *17* | Fusobacteria | *F*. *nucleatum* (21) |  |  |  |  |
|  | Actinobacteria | *Actinomyces* *lingae* (44), *Corynebacterium* *jeikeium* (336), *Atopobium* *rimae* (3338), *A*. *parvulum* (128) |  |  | Actinobacteria | *R*. *corynebacteroides* (1), *M*. *luteus* (1) |
|  | Bacteroidetes | *Prevotella* *denticola* (208), *P*. *oris* (3528), *Prevotella* *pallens* (338), *P*. *melaninogenica* (1156), *Prevotella* *ourulum* (40), *P*. *histicola* (66), *P*. *nigrescens* (202), *P*. *salivae* (230), *Prevotella* *veroralis* (250) |  |  |  |  |
|  | LAB | *S*. *constellatus* (235), *Enterococcus* *faecalis* (40), *Lactobacillus* *crispatus* (1758), *L*. *gasseri* (19) | LAB | *S*. *pneumoniae* (7) |  |  |
|  |  |  | Firmicutes | *S*. *haemolyticus* (6) | Firmicutes | *S*. *epidermidis* (1) |
| *18* |  |  | Enterobacteriaceae | *E*. *aerogenes* (13), *Klebsiella* spp. (2) |  |  |
|  |  |  |  |  | Actinobacteria | *R*. *corynebacteroides* (1) |
| *19* |  |  | Fusobacteria | *Leptotrichia* spp.(8) |  |  |
|  |  |  | β-Proteobacteria | *Alcaligenes* spp. (5) |  |  |
| *20* |  |  | LAB | *S*. *pneumoniae* (7) |  |  |
|  |  |  | Actinobacteria | *A.odontolyticus* (2) |  |  |
