# Supplementary material for: Assessment of Microbial Diversity in Biofilms Recovered from Endotracheal Tubes Using Culture Dependent and Independent Approaches
Source: PLoS One. 2012 Jun 5;7(6):e38401. doi: 10.1371/journal.pone.0038401 (PMC3367921; doi:10.1371/journal.pone.0038401)
Supplement: Table S3 — Simpson index of diversity (1-D) and Good’s coverage coefficients (C) of ET biofilms investigated by cultivation (cult), 16 S rRNA gene clone libraries (cl) and pyrosequencing of PCR amplified 16 S rRNA genes (pyro). (DOCX) [file pone.0038401.s004.docx]

| **Sample** | **1-D (cult)** | **1-D (cl)** | **1-D (pyro)** | **C (cult)** | **C (cl)** | **C (pyro)** |
| --- | --- | --- | --- | --- | --- | --- |
| **E1** | 0.79 | 0.34 | 0.51 | 65 | 90 | 99 |
| **E2** | 0.44 | 0.49 |  | 33 | 93 |  |
| **E3** | 0.75 | 0.52 |  | 0 | 93 |  |
| **E4** | 0.78 | 0.42 | 0.87 | 60 | 97 | 92 |
| **E5** | 0.79 | 0.59 |  | 38 | 95 |  |
| **E6** | 0.44 | 0 |  | 33 | 98 |  |
| **E7** | 0.72 | 0.12 |  | 33 | 91 |  |
| **E8** | 0.67 | 0.32 |  | 0 | 95 |  |
| **E9** | 0.5 | 0.38 |  | 0 | 91 |  |
| **E10** | 0.75 | 0.44 |  | 17 | 96 |  |
| **E11** | 0.56 | 0.58 |  | 50 | 97 |  |
| **E12** | 0.85 | 0.40 |  | 50 | 95 |  |
| **E13** | 0.64 | 0.53 | 0.81 | 62 | 94 | 94 |
| **E14** | 0.82 | 0.39 |  | 50 | 97 |  |
| **E15** | 0.29 | 0.38 |  | 75 | 96 |  |
| **E16** | 0.87 | 0.16 |  | 40 | 95 |  |
| **E17** | 0.86 | 0.32 | 0.83 | 38 | 98 | 89 |
| **E18** | 0.75 | 0.40 |  | 38 | 94 |  |
| **E19** | 0.63 | 0.40 |  | 25 | 96 |  |
| **E20** | 0.18 | 0.40 |  | 80 | 95 |  |
| **E21** | 0 |  |  | 93 |  |  |
| **E22** | 0.43 |  |  | 55 |  |  |
| **E23** | 0 |  |  | 93 |  |  |
| **E24** | 0.74 |  |  | 85 |  |  |
| **E25** | 0.27 |  |  | 25 |  |  |
| **E26** | 0.63 |  |  | 55 |  |  |
| **E27** | 0 |  |  | 66 |  |  |
| **E28** | 0 |  |  | 96 |  |  |
| **E29** | 0 |  |  | 50 |  |  |
| **E30** | 0.72 |  |  | 76 |  |  |
| **E31** | 0 |  |  | 50 |  |  |
| **E32** | 0.48 |  |  | 80 |  |  |
| **E33** | 0.47 |  |  | 75 |  |  |
| **E34** | 0.38 |  |  | 50 |  |  |
| **E35** | 0.44 |  |  | 66 |  |  |
| **E36** | 0 |  |  | 0 |  |  |
| **E37** | 0.37 |  |  | 79 |  |  |
| **E38** | 0 |  |  | 0 |  |  |
| **E39** | 0 |  |  | 80 |  |  |
| **E40** | 0.44 |  |  | 33 |  |  |
| **E41** | 0.49 |  |  | 89 |  |  |
| **E42** | 0.45 |  |  | 57 |  |  |
| **E43** | 0.18 |  |  | 87 |  |  |
| **E44** | 0.69 |  |  | 50 |  |  |
| **E45** | 0 |  |  | 83 |  |  |
| **E46** | 0.43 |  |  | 83 |  |  |
| **E47** | 0.44 |  |  | 67 |  |  |
| **E48** | 0 |  |  | 90 |  |  |
| **E49** | 0.44 |  |  | 78 |  |  |
| **E50** | 0.13 |  |  | 86 |  |  |
| **E51** | 0 |  |  | 88 |  |  |
| **E52** | 0.43 |  |  | 33 |  |  |
| **E53** | 0.44 |  |  | 57 |  |  |
| **E54** | 0.63 |  |  | 75 |  |  |
| **E55** | 0.17 |  |  | 82 |  |  |
