# Supplementary material for: Assessment of Microbial Diversity in Biofilms Recovered from Endotracheal Tubes Using Culture Dependent and Independent Approaches
Source: PLoS One. 2012 Jun 5;7(6):e38401. doi: 10.1371/journal.pone.0038401 (PMC3367921; doi:10.1371/journal.pone.0038401)
Supplement: Table S4 — Collector’s data of the first five clone libraries (cE1 to cE5). The cut-off for species delineation was 99% sequence similarity. OTU: operational taxonomic unit. (DOCX) [file pone.0038401.s005.docx]

| cE1 | | cE2 | | cE3 | | cE4 | | cE5 | |
| --- | --- | --- | --- | --- | --- | --- | --- | --- | --- |
| #clones | #OTUs | #clones | #OTUs | #clones | #OTUs | #clones | #OTUs | #clones | #OTUs |
| \| 1 \| 1 \| \| --- \| --- \| \| 2 \| 1 \| \| 3 \| 2 \| \| 4 \| 2 \| \| 5 \| 2 \| \| 6 \| 2 \| \| 7 \| 3 \| \| 8 \| 3 \| \| 9 \| 3 \| \| 10 \| 3 \| \| 11 \| 3 \| \| 12 \| 3 \| \| 13 \| 3 \| \| 14 \| 4 \| \| 15 \| 4 \| \| 16 \| 4 \| \| 17 \| 4 \| \| 18 \| 4 \| \| 19 \| 4 \| \| 20 \| 5 \| \| 21 \| 5 \| \| 22 \| 5 \| \| 23 \| 5 \| \| 24 \| 5 \| \| 25 \| 5 \| \| 26 \| 5 \| \| 27 \| 5 \| \| 28 \| 5 \| | | \| 1 \| 1 \| \| --- \| --- \| \| 3 \| 2 \| \| 4 \| 2 \| \| 5 \| 2 \| \| 6 \| 2 \| \| 7 \| 2 \| \| 8 \| 2 \| \| 9 \| 2 \| \| 10 \| 2 \| \| 11 \| 2 \| \| 12 \| 2 \| \| 13 \| 2 \| \| 14 \| 2 \| \| 15 \| 2 \| \| 16 \| 2 \| \| 17 \| 2 \| \| 18 \| 2 \| \| 19 \| 3 \| \| 20 \| 3 \| \| 21 \| 3 \| \| 22 \| 3 \| \| 23 \| 3 \| \| 24 \| 3 \| \| 25 \| 3 \| \| 26 \| 3 \| \| 27 \| 3 \| \| 28 \| 3 \| \| 29 \| 3 \| \| 30 \| 3 \| \| 31 \| 3 \| \| 32 \| 3 \| \| 33 \| 3 \| \| 34 \| 3 \| \| 35 \| 3 \| \| 36 \| 3 \| \| 37 \| 3 \| \| 38 \| 3 \| \| 39 \| 3 \| \| 40 \| 3 \| \| 41 \| 3 \| \| 42 \| 3 \| \| 43 \| 3 \| \| 44 \| 3 \| \| 45 \| 3 \| \| 46 \| 3 \| | | \| 1 \| 1 \| \| --- \| --- \| \| 2 \| 2 \| \| 3 \| 2 \| \| 4 \| 2 \| \| 5 \| 2 \| \| 6 \| 2 \| \| 7 \| 3 \| \| 8 \| 3 \| \| 9 \| 3 \| \| 10 \| 3 \| \| 11 \| 3 \| \| 12 \| 3 \| \| 13 \| 3 \| \| 14 \| 3 \| \| 15 \| 3 \| \| 16 \| 4 \| \| 17 \| 4 \| \| 18 \| 4 \| \| 19 \| 4 \| \| 20 \| 4 \| \| 21 \| 4 \| \| 22 \| 4 \| \| 23 \| 4 \| \| 24 \| 4 \| \| 25 \| 4 \| \| 26 \| 4 \| \| 27 \| 4 \| \| 28 \| 4 \| \| 29 \| 4 \| \| 30 \| 4 \| \| 31 \| 4 \| \| 32 \| 4 \| | | \| 1 \| 1 \| \| --- \| --- \| \| 2 \| 1 \| \| 3 \| 1 \| \| 4 \| 1 \| \| 5 \| 1 \| \| 6 \| 2 \| \| 7 \| 2 \| \| 8 \| 2 \| \| 9 \| 2 \| \| 10 \| 2 \| \| 11 \| 2 \| \| 12 \| 2 \| \| 13 \| 2 \| \| 14 \| 2 \| \| 15 \| 2 \| \| 16 \| 2 \| \| 17 \| 2 \| \| 18 \| 2 \| \| 19 \| 2 \| \| 20 \| 2 \| \| 21 \| 2 \| | | \| 1 \| 1 \| \| --- \| --- \| \| 2 \| 2 \| \| 3 \| 2 \| \| 4 \| 2 \| \| 5 \| 3 \| \| 6 \| 3 \| \| 7 \| 3 \| \| 8 \| 3 \| \| 9 \| 3 \| \| 10 \| 3 \| \| 11 \| 3 \| \| 12 \| 3 \| | |
